# Green synthesis of *Saussurea costus*-mediated silver nanoparticles: Characterization, antibacterial activity against ESBL-producing bacteria, and cytocompatibility

**DOI:** 10.1016/j.jgeb.2026.100794

**Published:** 2026-08-18

**Authors:** Rebwar Muhammad Hamasalih

**Affiliations:** Department of Biology, College of Education, Salahaddin University-Erbil, Erbil, 44002, Kurdistan Region, Iraq

**Keywords:** *Saussurea costus*, Silver nanoparticles, Green synthesis, ESBL-producing bacteria, Multidrug resistance, Cytocompatibility

## Abstract

The rapid emergence of extended-spectrum β-lactamase (ESBL)-producing *Escherichia coli* and *Klebsiella pneumoniae* has significantly limited the effectiveness of conventional antibiotics, highlighting the urgent need for alternative antimicrobial agents. In the present study, silver nanoparticles (AgNPs) were green synthesized using *Saussurea costus* root extract as a natural reducing and stabilizing agent. The biosynthesized AgNPs were comprehensively characterized by UV–visible spectroscopy, Fourier-transform infrared spectroscopy (FTIR), X-ray diffraction (XRD), transmission electron microscopy (TEM), dynamic light scattering (DLS), zeta potential analysis, and energy-dispersive X-ray spectroscopy (EDX), confirming the successful formation of stable, crystalline, predominantly spherical nanoparticles. TEM and DLS analyses demonstrated nanoscale particle dimensions, while the negative zeta potential indicated favorable colloidal stability.

The antibacterial activity of the synthesized AgNPs was evaluated against clinical ESBL-producing *E. coli* and *K. pneumoniae* isolates using agar well diffusion, minimum inhibitory concentration (MIC), minimum bactericidal concentration (MBC), and time-kill assays. The AgNPs exhibited pronounced concentration-dependent antibacterial activity, producing clear inhibition zones and low MIC and MBC values against both pathogens. Time-kill analysis further demonstrated rapid bactericidal activity, with increasing nanoparticle concentrations resulting in enhanced bacterial killing over time. Cytocompatibility evaluation using primary human dermal fibroblasts (HDFa) revealed acceptable cell viability within the antibacterial concentration range, supporting the preliminary biocompatibility of the biosynthesized nanoparticles.

Collectively, these findings demonstrate that *S. costus*-mediated AgNPs possess favorable physicochemical properties, potent in vitro antibacterial activity against clinically important ESBL-producing pathogens, and promising preliminary cytocompatibility. The study highlights the potential of *S. costus* as a sustainable plant source for green nanoparticle synthesis and provides a basis for the future development of plant-mediated antimicrobial nanomaterials. Nevertheless, additional investigations, including mechanistic studies, long-term stability assessment, comprehensive biosafety evaluation, and in vivo validation, are required before biomedical or clinical translation can be considered.

## Highlights

• Green synthesis: Stable, crystalline AgNPs were synthesized using Saussurea costus root extract as a natural reducing agent.
• Strong antibacterial activity: AgNPs showed potent activity against multidrug-resistant *E. coli* and *K. pneumoniae*.
• Rapid bactericidal effect: Time-kill assays showed enhanced bacterial killing over time at higher AgNP concentrations.
• Preliminary biocompatibility: AgNPs showed acceptable human dermal fibroblast viability at antibacterial concentrations.
• Promising alternative: *S. costus*-mediated AgNPs are sustainable antimicrobial candidates; further safety studies are needed.

## 1 Introduction

Antimicrobial resistance (AMR) has emerged as one of the most critical threats to global public health, substantially increasing morbidity, mortality, healthcare expenditures, and the complexity of clinical management. The rapid dissemination of multidrug-resistant (MDR) Gram-negative pathogens has significantly reduced the effectiveness of conventional antibiotic therapies, creating an urgent need for alternative antimicrobial strategies. Among these pathogens, extended-spectrum β-lactamase (ESBL)-producing Enterobacterales represent a major clinical concern because of their ability to hydrolyze a broad spectrum of β-lactam antibiotics while frequently harboring additional resistance determinants against aminoglycosides, fluoroquinolones, and sulfonamides.^1^, ^2^

Among ESBL-producing bacteria, *Escherichia coli* and *Klebsiella pneumoniae* are recognized as the predominant etiological agents of healthcare-associated and community-acquired infections worldwide. These organisms are responsible for urinary tract infections, bloodstream infections, pneumonia, wound infections, intra-abdominal infections, and sepsis, particularly among immunocompromised and hospitalized patients. Their remarkable capacity to acquire mobile genetic elements carrying blaCTX-M, blaTEM, and blaSHV genes has accelerated the global spread of antimicrobial resistance and severely limited therapeutic options.^3^

*Escherichia coli* remains the leading cause of urinary tract infections and is increasingly associated with bloodstream and surgical-site infections caused by ESBL-producing strains. The predominance of CTX-M-producing *E. coli* has transformed empirical treatment strategies, as many isolates exhibit resistance to third-generation cephalosporins, fluoroquinolones, and trimethoprim-sulfamethoxazole. Similarly, *Klebsiella pneumoniae* has become one of the most problematic opportunistic pathogens in healthcare settings owing to its extensive virulence repertoire, biofilm-forming ability, polysaccharide capsule, and rapid acquisition of plasmid-mediated resistance genes. The coexistence of ESBL production with carbapenem resistance has further complicated infection control and antimicrobial therapy worldwide.^4^

The growing prevalence of MDR *E. coli* and *K. pneumoniae* has stimulated considerable interest in nanotechnology-based antimicrobial agents. Among various nanomaterials, silver nanoparticles (AgNPs) have attracted particular attention because of their broad-spectrum antimicrobial activity, high surface-area-to-volume ratio, multiple bactericidal mechanisms, and reduced likelihood of inducing bacterial resistance. Unlike conventional antibiotics that target specific metabolic pathways, AgNPs disrupt bacterial membranes, generate reactive oxygen species, interfere with protein function, and damage nucleic acids through simultaneous mechanisms, thereby limiting the emergence of resistance.^5^, ^6^, ^7^

Recently, green synthesis has become the preferred strategy for producing AgNPs because it eliminates the use of hazardous reducing agents while employing naturally occurring phytochemicals as both reducing and stabilizing molecules.^8^ Recently, green synthesis has become the preferred strategy for producing AgNPs because it eliminates the use of hazardous reducing agents while employing naturally occurring phytochemicals as both reducing and stabilizing molecules.^8^ Green nanotechnology has emerged as a sustainable approach for nanoparticle synthesis, minimizing hazardous chemicals while improving environmental compatibility and biomedical applicability. Recent studies have further emphasized the role of eco-friendly nanoparticle synthesis in sustainable development and environmental protection.^9^

*Saussurea costus* (Falc.) Lipsch., a medicinal plant belonging to the family Asteraceae, has been extensively utilized in traditional medicine because of its broad spectrum of pharmacological properties, including antimicrobial, anti-inflammatory, antioxidant, anticancer, hepatoprotective, and immunomodulatory activities.^10^ The roots of *S. costus* are particularly rich in bioactive phytochemicals such as sesquiterpene lactones (costunolide and dehydrocostus lactone), flavonoids, alkaloids, tannins, phenolic compounds, and terpenoids, many of which possess strong reducing and metal-chelating capabilities that facilitate the biosynthesis and stabilization of metallic nanoparticles.^11^

The phytochemicals present in *S. costus* extract not only reduce silver ions (Ag^+^) into elemental silver (Ag^0^) but also adsorb onto the nanoparticle surface, acting as natural capping agents that improve colloidal stability, prevent aggregation, and enhance biological performance. Compared with chemically synthesized nanoparticles, plant-mediated AgNPs generally exhibit lower toxicity, improved biocompatibility, greater environmental sustainability, and enhanced biomedical applicability.^12^

Previous studies have demonstrated that *Saussurea costus*-mediated silver nanoparticles possess promising antimicrobial activity against a variety of bacterial pathogens and exhibit favorable physicochemical characteristics. However, these investigations have largely been limited to standard laboratory strains or preliminary antimicrobial screening, with relatively little emphasis on clinically relevant multidrug-resistant isolates. In particular, comprehensive studies evaluating *S. costus*-derived AgNPs against ESBL-producing *Escherichia coli* and *Klebsiella pneumoniae* clinical isolates, together with detailed characterization, bactericidal kinetics, antibiofilm activity, antioxidant potential, and cytocompatibility, remain scarce. This unmet need provided the primary rationale for the present investigation.^13^

The physicochemical characteristics of biosynthesized AgNPs, including particle size, morphology, crystallinity, surface chemistry, elemental composition, hydrodynamic diameter, and zeta potential, critically influence their biological activity. Therefore, comprehensive characterization using complementary analytical techniques such as UV–Visible spectroscopy, Fourier-transform infrared spectroscopy (FTIR), X-ray diffraction (XRD), field-emission scanning electron microscopy (FESEM), transmission electron microscopy (TEM), energy-dispersive X-ray spectroscopy (EDX), dynamic light scattering (DLS), and zeta potential analysis is essential to confirm successful nanoparticle synthesis and evaluate their structural stability.^14^

Despite the increasing number of reports describing the green synthesis of silver nanoparticles using medicinal plants, several important limitations remain. Most plant-mediated AgNP studies have focused on nanoparticle synthesis and routine physicochemical characterization, whereas systematic evaluation against clinically relevant multidrug-resistant pathogens has been limited. Moreover, direct comparisons among published studies are challenging because differences in plant extracts, synthesis conditions, nanoparticle physicochemical properties, and antimicrobial testng protocols frequently result in inconsistent biological outcomes. Consequently, it remains unclear whether biosynthesized AgNPs derived from different medicinal plants possess comparable antibacterial efficacy or sufficient biological safety for potential biomedical applications. In addition, although *Saussurea costus* has been recognized as a promising source of reducing and stabilizing phytochemicals, only a few studies have investigated its AgNPs beyond preliminary antimicrobial screening, and comprehensive assessments integrating antibacterial efficacy, antioxidant activity, bactericidal kinetics, biofilm inhibition, and cytocompatibility remain scarce. Recent reviews have highlighted the need for standardized biological evaluation and mechanistic studies to facilitate the clinical translation of plant-mediated AgNPs.^11^

Another important unresolved issue concerns the relationship between the physicochemical characteristics of biosynthesized AgNPs and their biological performance. Although particle size, morphology, surface chemistry, and surface charge are recognized as critical determinants of antimicrobial activity, the mechanisms by which phytochemical capping agents derived from *S. costus* influence nanoparticle stability, antibacterial efficacy, and mammalian cell compatibility remain incompletely understood. Furthermore, no previous study has comprehensively evaluated *S. costus*-derived AgNPs against clinically isolated ESBL-producing *Escherichia coli* and *Klebsiella pneumoniae* while simultaneously assessing antibacterial activity, MIC/MBC, time-kill kinetics, antibiofilm activity, antioxidant capacity, and cytocompatibility using normal human dermal fibroblasts. This represents an important knowledge gap that limits the translational assessment of these biosynthesized nanoparticles. Based on the phytochemical composition of *S. costus*, we hypothesized that biosynthesized AgNPs would exhibit favorable physicochemical stability together with potent antibacterial activity against clinical ESBL-producing isolates while maintaining acceptable cytocompatibility toward normal mammalian cells.^15^

Although several studies have reported the green synthesis of silver nanoparticles using *Saussurea costus*, most investigations have focused primarily on nanoparticle synthesis and preliminary antimicrobial evaluation against standard laboratory strains. Limited information is available regarding their efficacy against clinically isolated ESBL-producing multidrug-resistant pathogens together with comprehensive physicochemical characterization, antioxidant activity, bactericidal kinetics, and cytocompatibility evaluation. Therefore, the present study aimed to address this gap.

To test this hypothesis, silver nanoparticles were biosynthesized using aqueous root extract of *Saussurea costus* through an environmentally friendly approach and comprehensively characterized using complementary physicochemical techniques. Their antibacterial activity was subsequently evaluated against clinically isolated ESBL-producing *Escherichia coli* and *Klebsiella pneumoniae* through antimicrobial susceptibility testing, MIC/MBC determination, time-kill kinetics, and antibiofilm assays. In addition, antioxidant activity and cytocompatibility using human dermal fibroblast adult (HDFa) cells were investigated to provide an integrated preclinical assessment of the biosynthesized nanoparticles and their potential biomedical applicability.

## 2 Material and methods

### 2.1 Plant mate rial collection and authentication

Fresh roots of *Saussurea costus* (Falc.) Lipsch. were harvested during the flowering period (June–July 2024) from mountainous areas in Shaqlawa, Erbil Governorate, Kurdistan Region, Iraq (36°24′N, 44°19′E; elevation ∼950–1100 m above sea level). Only healthy, fully mature specimens free from disease symptoms, fungal contamination, insect damage, or mechanical injury were selected based on morphological examination. Immediately following harvest, plant materials were transferred into sterile Whirl-Pak® polyethylene bags (Nasco, Fort Atkinson, WI, USA) and transported to the Microbiology Research Laboratory within 2 h under ambient conditions.^16^

Taxonomic authentication of the collected specimens was carried out by a qualified plant taxonomist from the Department of Biology, College of Science, Salahaddin University-Erbil, Erbil, Iraq, utilizing taxonomic keys from the Flora of Iraq. A voucher specimen (Voucher No. SC-2024-017) was prepared and deposited in the Herbarium of the College of Science, Salahaddin University-Erbil for future reference.

Roots were thoroughly cleaned under running tap water to eliminate adhering soil and debris, followed by three sequential washes with sterile distilled water. The cleaned roots were sectioned into small pieces (2–3 cm) using sterile stainless-steel scissors and subjected to shade drying at room temperature (25 ± 2 °C) with continuous ventilation for 14 days until constant weight was achieved. Direct sunlight exposure was carefully avoided to prevent degradation of thermolabile and photosensitive phytochemicals, particularly phenolic compounds, flavonoids, and sesquiterpene lactones known to participate in metal nanoparticle reduction and stabilization.

The completely desiccated roots were ground to a fine powder using an IKA® A11 Basic Analytical Mill (IKA-Werke GmbH & Co. KG, Staufen, Germany). The powdered material was sieved through a sterile 60-mesh stainless-steel sieve (250 μm) to achieve uniform particle size distribution and subsequently stored in sterile amber glass containers at room temperature (22–25 °C) in a dry, dark environment pending extraction.

Strict aseptic techniques were maintained throughout sample preparation to preserve phytochemical integrity and prevent microbial contamination. All glassware was cleaned with laboratory-grade detergent, rinsed with distilled water, and sterilized in a hot-air oven at 160 °C for 2 h. Heat-stable materials were autoclaved at 121 °C for 15 min under 15 psi pressure. Stainless-steel instruments were disinfected with 70% (*v*/v) ethanol before each use. All procedures were conducted under standard laboratory conditions to ensure reproducibility of subsequent green synthesis experiments.

### 2.2 Preparation of *Saussurea costus* root aqueous extract

Dried *S. costus* roots were pulverized using an electric laboratory grinder and passed through a sterile 60-mesh sieve to obtain a homogeneous powder. The powdered material was kept in airtight amber glass bottles at room temperature until extraction.

For aqueous extraction, 10 g of dried root powder was accurately weighed using a calibrated Sartorius Entris II analytical balance (Sartorius AG, Göttingen, Germany) and transferred to a sterile 250-mL borosilicate Erlenmeyer flask containing 100 mL of ultrapure deionized water (18.2 MΩ·cm, Milli-Q, Millipore, Bedford, MA, USA). The mixture was heated at 80 °C for 20 min on a digital hot-plate magnetic stirrer (IKA C-MAG HS7, IKA-Werke GmbH & Co. KG, Staufen, Germany) with continuous stirring at 400 rpm to facilitate extraction of water-soluble phytochemicals, including phenolic compounds, flavonoids, terpenoids, alkaloids, reducing sugars, and sesquiterpene lactones, which serve as natural reducing and stabilizing agents during nanoparticle synthesis.

Following heating, the extract was allowed to cool to room temperature (25 ± 2 °C) and incubated for an additional 30 min to maximize bioactive constituent diffusion into the aqueous phase. The resulting suspension was filtered twice through sterile Whatman No. 1 filter paper (GE Healthcare Life Sciences, Buckinghamshire, UK) to remove coarse debris. The filtrate was then centrifuged at 5000 ×*g* for 15 min at 4 °C using a refrigerated Eppendorf 5810R centrifuge (Hamburg, Germany) to eliminate fine suspended particles and obtain a clear aqueous extract. The supernatant was carefully collected under aseptic conditions.

The clarified aqueous extract was transferred to sterile amber glass bottles and stored at 4 °C until use. To preserve phytochemical reducing activity and minimize oxidative degradation, all extracts were utilized within 48 h of preparation. Fresh extract was prepared whenever reduced efficiency or color/turbidity changes were observed.

All extraction procedures were performed under sterile conditions using autoclaved glassware to prevent microbial contamination. Three independent extractions were performed, and freshly prepared extracts from each batch were used for silver nanoparticle biosynthesis to ensure experimental reproducibility.

### 2.3 Green synthesis of silver nanoparticles

Silver nanoparticles (AgNPs) were synthesized using *S. costus* aqueous root extract via a green reduction approach with modifications to established protocols. Briefly, a 1 mM aqueous silver nitrate (AgNO₃) solution was freshly prepared by dissolving 0.01699 g of analytical-grade AgNO₃ (Sigma–Aldrich, St. Louis, MO, USA; ≥99.9% purity) in 100 mL of ultrapure deionized water. The solution was prepared immediately before each experiment and protected from light using aluminum foil to prevent photoreduction.

For nanoparticle synthesis, 10 mL of freshly prepared *S. costus* aqueous root extract was added dropwise to 100 mL of the AgNO₃ solution under continuous magnetic stirring (400 rpm) using a digital magnetic stirrer (IKA C-MAG HS7, IKA-Werke GmbH, Germany). The reaction mixture was maintained at room temperature (27 ± 2 °C) at pH 7.0 ± 0.2 without external reducing or stabilizing agents. The mixture was incubated for 24 h under continuous shaking (150 rpm) in an orbital incubator shaker (New Brunswick Innova 42, Eppendorf, Germany) under dark conditions.

Reaction progress was monitored both visually and spectrophotometrically. A gradual color change from pale yellow to dark brown indicated AgNP formation due to localized surface plasmon resonance (SPR) excitation. Aliquots (1 mL) were aseptically withdrawn at predetermined intervals (0, 1, 3, 6, 12, and 24 h) for UV–Visible spectroscopic monitoring.

Upon reaction completion, the suspension was centrifuged at 12,000 ×*g* for 20 min at 4 °C (Eppendorf 5810R, Hamburg, Germany). The supernatant containing residual phytochemicals and unreacted silver ions was discarded, and the nanoparticle pellet was washed three times with sterile deionized water followed by one wash with absolute ethanol. Each washing step was followed by centrifugation under identical conditions.

The purified AgNP pellet was dried in a vacuum oven (Binder VD23, Tuttlingen, Germany) at 40 °C for 24 h until constant weight. The dried nanoparticles were gently ground using a sterile agate mortar and pestle to obtain a homogeneous powder and stored in sterile amber glass vials at 4 °C until characterization and biological assays.

For antibacterial experiments, a sterile AgNP stock suspension (1 mg/mL) was prepared in ultrapure water and dispersed by ultrasonication (Branson Ultrasonics 2800, Emerson, USA) for 15 min (40 kHz, 100 W) immediately before each experiment. The suspension was sterilized by UV exposure in a Class II biological safety cabinet for 30 min before biological applications.

Three independent nanoparticle synthesis batches were prepared under identical conditions to assess reproducibility. Each batch was characterized separately before pooling for biological experiments. Control experiments (AgNO₃ solution without plant extract and plant extract without AgNO₃) were processed simultaneously under identical conditions to confirm phytochemical-mediated reduction.

#### 2.3.1 UV–visible spectroscopic analysis

The formation and optical characteristics of biosynthesized AgNPs were initially confirmed by UV–Visible spectroscopy based on the surface plasmon resonance (SPR) phenomenon, a reliable preliminary indicator of silver nanoparticle formation.^16^

Spectral analysis was performed using a UV-1800 spectrophotometer (Shimadzu Corporation, Kyoto, Japan) equipped with a deuterium–halogen light source and controlled by UVProbe software (Version 2.70). Wavelength calibration was verified before each measurement per manufacturer specifications.

For analysis, 1 mL of freshly synthesized AgNP suspension (after 24 h incubation) was diluted ten-fold with ultrapure deionized water to minimize scattering effects and maintain absorbance within the linear detection range. The diluted suspension was vortexed for 30 s and sonicated for 5 min (Branson Ultrasonics 2800, Emerson, USA; 40 kHz) to ensure homogeneous dispersion before measurement.^17^

Absorbance spectra were recorded over 300–700 nm using matched 1-cm path-length quartz cuvettes. Ultrapure deionized water served as the blank reference, and each spectrum represented the average of three consecutive scans at 300 nm min^−1^ with 1 nm resolution. Baseline correction was automatically performed before each analysis.

To monitor nanoparticle formation kinetics, UV–Visible spectra were recorded at different reaction times (0, 1, 3, 6, 12, and 24 h). The gradual increase in absorbance intensity with the appearance of a characteristic SPR absorption band (approximately 400–460 nm) indicated reduction of Ag^+^ to metallic Ag^0^ nanoparticles. The wavelength of maximum absorption (λ_max_) was determined automatically and compared with published values. All measurements were performed in triplicate at room temperature (25 ± 2 °C) using three independently synthesized nanoparticle batches. Results were expressed as mean absorbance ± standard deviation.

UV–Visible spectra served as preliminary quality control before advanced characterization by FTIR, XRD, SEM, TEM, DLS, and zeta potential analysis. Optical properties were interpreted according to SPR theory, where conduction-band electron oscillation interacts with incident electromagnetic radiation. Variations in SPR peak position, width, and intensity indicated changes in nanoparticle size distribution, morphology, concentration, and colloidal stability.

#### 2.3.2 Fourier transform infrared (FTIR) spectroscopy

FTIR spectroscopy was employed to identify functional groups on biosynthesized AgNP surfaces and determine phytochemical constituents of *S. costus* root extract involved in silver ion reduction and nanoparticle stabilization.

Prior to FTIR analysis, purified AgNP suspension was dried in a vacuum oven (Binder VD23, Binder GmbH, Tuttlingen, Germany) at 40 °C for 24 h. Dried nanoparticles were finely powdered using a sterile agate mortar and pestle and stored in a desiccator with silica gel until analysis.

FTIR spectra were recorded using an IRTracer-100 spectrometer (Shimadzu Corporation, Kyoto, Japan) with a deuterated triglycine sulfate (DTGS) detector and attenuated total reflectance (ATR) accessory with diamond crystal. Instrument performance was verified using a polystyrene reference standard.

Approximately 2–3 mg of dried AgNP powder was placed on the ATR crystal and compressed to ensure complete contact. Spectra were collected over 4000–400 cm^−1^ at 4 cm^−1^ resolution with 32 cumulative scans. Background spectra were recorded before each sample and automatically subtracted to eliminate atmospheric interference.

FTIR spectra of both freeze-dried aqueous root extract and purified AgNPs were recorded under identical conditions for comparative analysis. Peak shifts, intensity changes, and disappearance/appearance of absorption bands following nanoparticle formation indicated interactions between phytochemical functional groups and AgNP surfaces.

Peak assignment was performed using Shimadzu IRsolution software (Version 1.60) and published spectral databases. Characteristic absorption bands corresponding to hydroxyl (–OH), amine (–NH), carbonyl (C=O), carboxyl (–COOH), aromatic C=C, ether (C–O–C), phenolic C—O, and alkyl C—H stretching vibrations were identified. The presence of these functional groups indicated phenolic compounds, flavonoids, proteins, polysaccharides, terpenoids, and sesquiterpene lactones adsorbed onto nanoparticle surfaces as natural reducing and stabilizing agents.^11^

Each sample was analyzed in triplicate using three independently synthesized nanoparticle batches. Spectra were normalized and baseline-corrected before interpretation, and only consistent peaks observed in all independent analyses were considered.

FTIR analysis provided complementary molecular evidence supporting the phytochemical-mediated reduction mechanism and was interpreted alongside UV–Visible spectroscopy, XRD, FESEM, TEM, DLS, and zeta potential analyses for comprehensive physicochemical characterization.

#### 2.3.3 X-ray diffraction (XRD) analysis

The crystalline structure, phase purity, crystallographic orientation, and average crystallite size of biosynthesized AgNPs were investigated using powder X-ray diffraction (XRD). This technique confirms successful reduction of Ag^+^ into crystalline metallic Ag^0^ and distinguishes metallic silver from silver oxide or other crystalline impurities.

Purified AgNPs obtained after repeated washing and centrifugation were dried in a vacuum oven (Binder VD23, Binder GmbH, Tuttlingen, Germany) at 40 °C for 24 h. Dried nanoparticles were gently ground using an agate mortar and pestle and stored in sterile airtight glass vials with silica gel desiccant.

XRD measurements were performed using an XRD-6100 diffractometer (Shimadzu Corporation, Kyoto, Japan) with Cu-Kα radiation (λ = 1.5406 Å) at 40 kV and 30 mA. Instrument calibration was verified using a certified silicon standard.

Approximately 100 mg of finely powdered AgNPs was uniformly spread onto a low-background quartz sample holder and gently pressed to obtain a flat analytical surface while minimizing preferred crystal orientation.

Diffraction data were collected over a 2θ range of 20°–80°, encompassing principal diffraction peaks characteristic of face-centered cubic (fcc) metallic silver.^18^ Measurements were performed at 2° min^−1^ scanning speed, 0.02° step size, and 1 s counting time per step.

Raw diffraction patterns were processed using Shimadzu XRD-6000 Software with background subtraction and baseline correction. Diffraction peaks were identified by comparison with ICDD Powder Diffraction File (PDF Card No. 04–0783) for metallic silver.^18^

Diffraction peaks corresponding to crystallographic planes indexed as (111), (200), (220), and (311) confirmed crystalline fcc silver nanoparticle formation. Additional peaks were evaluated for residual phytochemicals or crystalline impurities.

Average crystallite size (D) was estimated using the Debye–Scherrer equation:

$$D=\mathrm{K\lambda}/\left(\beta \;\cos\;\theta \right).$$

where D is crystallite size (nm), K is the Scherrer shape factor (0.9), λ is X-ray wavelength (1.5406 Å), β is FWHM of the most intense diffraction peak in radians after instrumental broadening correction, and θ is the corresponding Bragg angle.

The (111) diffraction plane, typically exhibiting the highest intensity, was selected for crystallite size determination. Calculations were independently performed for three synthesis batches, with results expressed as mean ± standard deviation.

Lattice parameters were calculated using Bragg's law and fcc crystal structure equations, with the calculated lattice constant compared to bulk metallic silver standards.

Crystallinity was assessed by comparing diffraction peak sharpness and relative intensity with published patterns of biologically synthesized AgNPs. Broader peaks indicated nanoscale crystallites, while narrow, intense peaks suggested improved crystallinity.

XRD analysis was performed using three independently synthesized nanoparticle batches, with each sample scanned twice. Representative patterns were selected after confirming consistency.

Crystallographic information was correlated with FESEM and TEM morphology, DLS hydrodynamic diameter, and UV–Visible optical properties for comprehensive physicochemical characterization.

#### 2.3.4 Field emission scanning electron microscopy (FESEM)

Surface morphology, particle size distribution, agglomeration degree, and surface topography of biosynthesized AgNPs were examined using Field Emission Scanning Electron Microscopy (FESEM), providing high-resolution images with minimal electron beam-induced damage.

Purified AgNPs obtained after repeated washing and centrifugation were vacuum-dried at 40 °C for 24 h. Approximately 2 mg of dried powder was dispersed in 5 mL of absolute ethanol (≥99.9%, Merck, Darmstadt, Germany) and ultrasonicated for 15 min (Branson Ultrasonics 2800, Emerson, Danbury, CT, USA; 40 kHz, 100 W) to obtain a homogeneous suspension.

A droplet (10 μL) of the well-dispersed suspension was deposited onto a clean silicon wafer (previously washed with ethanol and acetone) and dried at room temperature under dust-free conditions. Dried samples were mounted on aluminum specimen stubs using conductive carbon adhesive tape and coated with an ∼8–10 nm gold layer using a sputter coater (Quorum Technologies Q150R ES, Lewes, United Kingdom) under argon for approximately 90 s.

Morphological analysis was performed using a FESEM (TESCAN MIRA3, TESCAN ORSAY HOLDING, Brno, Czech Republic) at 10–15 kV accelerating voltage with optimized beam current. Images were acquired under high-vacuum mode at working distances of 8–10 mm.

Micrographs were recorded at magnifications of 20,000× to 200,000× to evaluate overall nanoparticle distribution and individual particle morphology. Several randomly selected fields from different specimen regions were examined to minimize sampling bias.

Digital FESEM images were exported in TIFF format and analyzed using ImageJ software (National Institutes of Health, Bethesda, MD, USA; Version 1.54). The software was calibrated using the microscope scale bar. At least 200 individual nanoparticles from randomly selected fields were manually measured for particle diameter.

Particle size distribution was expressed as mean diameter ± standard deviation, with observed size range. Histogram analysis was performed using ImageJ for frequency distribution. Morphological descriptors including circularity, aspect ratio, and aggregation degree were assessed when image quality permitted.

Particular attention was given to morphological characteristics commonly associated with green-synthesized AgNPs, including spherical, quasi-spherical, oval, and occasionally irregular nanoparticles. Clustered particles were interpreted with caution due to potential drying artifacts or phytochemical capping agent interactions.

Surface texture was evaluated qualitatively to determine whether synthesized AgNPs possessed smooth or rough surfaces, indicating adsorption of *S. costus*-derived phytochemicals (phenolic compounds, flavonoids, terpenoids, sesquiterpene lactones) functioning as natural reducing and stabilizing agents.

FESEM analysis was performed using three independently synthesized nanoparticle batches, with multiple specimens from each batch. Representative micrographs were selected after confirming consistent morphological characteristics.

Particle size from FESEM was compared with crystallite size from XRD, core particle size from TEM, and hydrodynamic diameter from DLS, with differences interpreted according to measurement principles.

FESEM observations provided complementary morphological evidence supporting successful AgNP biosynthesis.

#### 2.3.5 Energy-dispersive X-ray spectroscopy (EDX) analysis

Elemental composition and chemical purity of biosynthesized AgNPs were investigated using Energy-Dispersive X-ray Spectroscopy (EDX) coupled with FESEM. EDX analysis confirmed Ag^+^ reduction to elemental Ag^0^, determined elemental abundance, identified impurities, and evaluated phytochemical capping molecules on nanoparticle surfaces.

Elemental analysis was conducted using an Oxford Instruments X-Max 80 mm^2^ Silicon Drift Detector (SDD) integrated with the TESCAN MIRA3 FESEM, with energy resolution ∼127 eV at Mn Kα, allowing accurate identification of characteristic X-ray emission peaks.^18^

Sample preparation for EDX was identical to FESEM observations. Approximately 2 mg of dried AgNP powder was ultrasonically dispersed in absolute ethanol for 15 min, deposited onto a clean silicon wafer, air-dried, mounted on aluminum stubs with carbon adhesive tape, and sputter-coated with gold.

Elemental spectra were acquired under high-vacuum conditions at 15 kV accelerating voltage with optimized probe current and ∼ 10 mm working distance. Detector calibration was verified using a certified copper standard. Instrument dead time was maintained between 20% and 35%.

For each nanoparticle batch, EDX spectra were collected from at least ten randomly selected microscopic fields. Each acquisition was performed for 60 s live counting time. Multiple spectra from different regions were averaged for representative elemental composition.

Qualitative elemental identification was performed using Oxford Aztec software (Version 4.3) by comparing detected characteristic X-ray emission energies with reference databases. Quantitative analysis was performed using ZAF correction for atomic number, absorption, and fluorescence effects.

Special attention was given to the characteristic silver (Ag) emission peak near 3.0 keV, the principal fingerprint of metallic silver nanoparticles. An intense Ag peak with absence of contaminating heavy metal peaks indicated successful high-purity AgNP biosynthesis.

In addition to silver, low-intensity signals corresponding to carbon (C), oxygen (O), and occasionally chlorine (Cl) were expected. Carbon and oxygen peaks indicated phytochemical constituents of *S. costus* adsorbed onto nanoparticle surfaces (phenolic compounds, flavonoids, carbohydrates, proteins, terpenoids). Minor chlorine signals were attributed to residual chloride ions rather than nanoparticle structural components. Gold peaks from sputter coating were excluded from quantitative interpretation.

Elemental mapping analysis evaluated spatial distribution of silver throughout analyzed regions. Two-dimensional distribution maps for Ag, C, and O were generated using the Aztec mapping module. Homogeneous silver distribution indicated uniform nanoparticle synthesis and successful Ag^+^ reduction.

Quantitative elemental composition was expressed as weight percentage (Wt%) and atomic percentage (At.%). Measurements were performed using three independently synthesized nanoparticle batches, with each analysis repeated three times. Results were reported as mean ± standard deviation.

EDX information was interpreted alongside FESEM morphology, FTIR functional group analysis, XRD crystallographic characterization, TEM particle size measurements, and DLS hydrodynamic diameter for comprehensive physicochemical profiling.

#### 2.3.6 Transmission electron microscopy (TEM) analysis

Transmission Electron Microscopy (TEM) was employed to investigate internal morphology, particle size, size distribution, structural uniformity, and dispersion behavior of biosynthesized AgNPs. With nanometer-scale spatial resolution, TEM is the reference technique for direct visualization of metallic nanoparticles and accurate measurement of primary particle diameter independent of hydrodynamic shell measured by DLS.

Purified AgNPs obtained after repeated centrifugation and washing were vacuum-dried at 40 °C for 24 h. Approximately 1 mg of dried powder was dispersed in 10 mL of ultrapure deionized water. The suspension was sonicated (Branson Ultrasonics 2800, Emerson, Danbury, CT, USA) at 40 kHz and 100 W for 20 min to disrupt weak agglomerates.^19^

Immediately after sonication, 10 μL of nanoparticle suspension was deposited onto a carbon-coated copper grid (300 mesh) (Electron Microscopy Sciences, Hatfield, PA, USA). Excess liquid was carefully removed using filter paper without touching the deposit. Grids were dried under dust-free conditions at room temperature for ∼12 h. No staining agents were applied as metallic silver nanoparticles possess sufficient electron density for intrinsic contrast.

TEM observations were carried out using a JEOL JEM-2100 Transmission Electron Microscope (JEOL Ltd., Tokyo, Japan) at 200 kV. The microscope was calibrated using a certified gold nanoparticle standard. Images were acquired using a high-resolution digital CCD camera under identical exposure conditions.

Nanoparticles were examined at magnifications of 50,000× to 400,000×. Images were acquired from randomly selected fields across different grid areas to minimize sampling bias. Regions with extensive overlap or excessive aggregation were excluded.

Particle size measurements were performed using ImageJ software (National Institutes of Health, Bethesda, MD, USA; Version 1.54). Image calibration was performed using the microscope scale bar. At least 200 individual nanoparticles from three independently synthesized batches were manually measured for primary particle diameter.

Particle size distribution was expressed as mean diameter ± standard deviation with minimum and maximum observed diameters. Frequency distribution histograms were generated using ImageJ. Coefficient of variation (CV) was calculated to assess size homogeneity.

Morphological evaluation included assessment of particle geometry, surface smoothness, aggregation state, and dispersion quality. Nanoparticles were classified as spherical, quasi-spherical, oval, polygonal, triangular, or irregular. The percentage of each morphological class was estimated qualitatively.

Particular attention was given to identifying a thin organic corona surrounding individual nanoparticles. This low-contrast peripheral layer, when observed, indicated phytochemical molecules from *S. costus* root extract adsorbed onto nanoparticle surfaces, contributing to stabilization through steric and electrostatic repulsion.

Selected Area Electron Diffraction (SAED) patterns were acquired from representative nanoparticle clusters. Concentric diffraction rings indexed to (111), (200), (220), and (311) crystallographic planes indicated polycrystalline fcc metallic silver structure. Diffraction rings were compared with JCPDS Card No. 04–0783.

TEM analysis was performed using three independently synthesized AgNP batches, with multiple grids from each batch. Representative images were selected after confirming consistency in nanoparticle morphology, particle size, and dispersion behavior [25,26].

Average particle diameter from TEM was compared with crystallite size from XRD and hydrodynamic diameter from DLS. Larger DLS values were attributed to hydration layer and adsorbed phytochemicals surrounding nanoparticles in aqueous suspension, while TEM provided direct measurement of metallic nanoparticle core.

TEM served as a complementary high-resolution imaging technique confirming successful biosynthesis of predominantly spherical, well-dispersed crystalline silver nanoparticles with nanoscale dimensions suitable for biological applications.

#### 2.3.7 Dynamic light scattering (DLS) analysis

Hydrodynamic diameter, particle size distribution, and colloidal homogeneity of biosynthesized AgNPs were determined using Dynamic Light Scattering (DLS). Unlike electron microscopy measuring physical core diameter of dried nanoparticles, DLS determines hydrodynamic diameter of dispersed nanoparticles including hydration layer and phytochemical corona, providing information on nanoparticle behavior under physiological conditions.

Purified AgNPs were dispersed in ultrapure deionized water at 0.1 mg/mL final concentration. The suspension was vortexed for 30 s and sonicated (Branson Ultrasonics 2800, Emerson, Danbury, CT, USA) at 40 kHz and 100 W for 15 min to minimize reversible aggregation.

The suspension was filtered through a 0.45 μm sterile syringe filter (Millipore, USA) when visible dust particles were present. No membrane filtration was applied if expected to remove larger nanoparticle aggregates.^20^

Particle size measurements were performed using a Zetasizer Nano ZS90 (Malvern Panalytical Ltd., Worcestershire, United Kingdom) with a 4 mW He—Ne laser (λ = 633 nm) and fixed 90° backscattering detection angle.

Disposable polystyrene cuvettes were rinsed three times with ultrapure water. Approximately 1 mL of nanoparticle suspension was transferred into each cuvette, ensuring complete absence of air bubbles.

Measurements were performed at 25 ± 0.1 °C controlled by an integrated Peltier temperature regulation system. Samples were equilibrated in the instrument chamber for 120 s to achieve thermal equilibrium.

Viscosity and refractive index of ultrapure water at 25 °C were entered as 0.8872 cP and 1.330, respectively. Refractive index of silver nanoparticles was set to 0.135 per manufacturer recommendations.

Each sample was analyzed using 12 consecutive measurement runs (15 sub-runs each), with average values automatically calculated using cumulant analysis in Malvern Zetasizer Software Version 8.02.

Hydrodynamic diameter was calculated from translational diffusion coefficient using the Stokes–Einstein equation:

$$\mathrm{DH}=\mathrm{kBT}/\left(\text{3πηD}\right)$$

where DH is hydrodynamic diameter, kB is Boltzmann constant, T is absolute temperature, η is dynamic viscosity of dispersing medium, and D is translational diffusion coefficient.^21^

Polydispersity Index (PDI) was simultaneously determined to evaluate particle size distribution. PDI values below 0.30 indicated relatively homogeneous nanoparticle populations, while larger values suggested broader distributions and possible aggregation. Three independently synthesized nanoparticle batches were analyzed, with each measurement repeated three times using freshly prepared suspensions. Results were expressed as mean ± standard deviation.

DLS results were interpreted alongside TEM observations. Differences between TEM particle size and DLS hydrodynamic diameter were attributed to hydration shell and adsorbed phytochemical molecules. DLS analysis provided essential information on nanoparticle dispersion behavior and colloidal characteristics under aqueous conditions, supporting suitability for biological applications.

### 2.4 Collection, identification, and characterization of clinical bacterial isolates

A total of 300 non-duplicate clinical bacterial isolates were prospectively collected from patients referred to major tertiary-care hospitals in Erbil, Kurdistan Region, Iraq, between January and December 2023. The study population included hospitalized and outpatient individuals with clinically suspected bacterial infections. Only the first isolate from each patient during the study period was included to eliminate duplicate sampling.^22^

Clinical specimens included urine, wound swabs, blood, sputum, tracheal aspirates, burn wound swabs, catheter tips, and other clinically significant samples submitted for routine diagnostic procedures. Specimens were collected by trained healthcare professionals using sterile techniques per standard clinical microbiology guidelines.

All specimens were transported to the Clinical Microbiology Laboratory under appropriate biosafety conditions and processed within 2 h to preserve bacterial viability. Samples not processed immediately were temporarily stored at 4 °C for no longer than 24 h per CLSI recommendations.

Primary bacterial isolation was performed by inoculating specimens onto Blood Agar, MacConkey Agar, and Chocolate Agar (Oxoid Ltd., Basingstoke, United Kingdom) using standard streak plate technique. Inoculated media were incubated aerobically at 37 ± 1 °C for 18–24 h, with extended 48-h incubation for slow-growing organisms when required.

Following incubation, bacterial colonies were evaluated for morphology, pigmentation, size, hemolytic activity, lactose fermentation, odor, and mucoid phenotype. Preliminary identification was based on conventional microbiological methods including Gram staining, oxidase testing, catalase testing, and standard biochemical characterization.

Species-level identification was confirmed using the VITEK® 2 Compact automated identification system (bioMérieux, Marcy-l'Étoile, France) with GN identification cards per manufacturer instructions. Quality assurance was performed daily using reference quality-control strains.

From the 300 bacterial isolates, only *Escherichia coli* and *Klebsiella pneumoniae* isolates were included in the present investigation. Other species recovered during routine microbiological examination were excluded. Pure cultures of confirmed isolates were maintained on Mueller–Hinton Agar (Oxoid, UK) and subcultured every two weeks. For long-term preservation, isolates were suspended in Tryptic Soy Broth (TSB) with 20% sterile glycerol (*v*/v) and stored at −80 °C.

Before each experiment, frozen isolates were revived on Mueller–Hinton Agar and incubated at 37 °C for 18–24 h. Fresh overnight cultures were used for all antibacterial susceptibility testing and biofilm assays.

Reference quality-control strains (*E. coli* ATCC 25922 and *K. pneumoniae* ATCC 700603) were included throughout for verification of bacterial identification, antimicrobial susceptibility testing, and phenotypic ESBL detection per CLSI recommendations. All laboratory procedures involving clinical isolates were conducted in a certified biosafety level 2 (BSL-2) microbiology laboratory using appropriate aseptic techniques and personal protective equipment

The study protocol was approved by the Research Ethics Committee of Erbil University of Medical Sciences, Erbil, Iraq, and all microbiological investigations were performed in accordance with the Declaration of Helsinki and institutional biosafety regulations. Patient confidentiality was maintained by assigning unique laboratory identification codes to each isolate.

### 2.5 Phenotypic detection of extended-spectrum β-lactamase (ESBL)-producing *Escherichia coli* and *Klebsiella pneumoniae*

Following species identification, all confirmed *E. coli* and *K. pneumoniae* isolates were screened for ESBL production using a two-step phenotypic approach (initial screening followed by confirmatory testing) per CLSI M100 (2024) recommendations.^23^

Each isolate was subcultured twice on Mueller–Hinton Agar (Oxoid Ltd., Basingstoke, United Kingdom) and incubated aerobically at 37 ± 1 °C for 18–24 h to ensure purity and optimal activity. Fresh overnight colonies were selected for inoculum preparation.

A standardized bacterial suspension equivalent to 0.5 McFarland turbidity (∼1.5 × 10^8^ CFU mL^−1^) was prepared by suspending three to five morphologically similar colonies in sterile physiological saline (0.85% NaCl). Turbidity was visually adjusted and verified using a DensiCHEK Plus densitometer (bioMérieux, Marcy-l'Étoile, France).

Within 15 min of preparation, a sterile cotton swab was immersed in the bacterial suspension, rotated, and pressed against the tube wall to remove excess liquid. Mueller–Hinton Agar plates were uniformly inoculated by streaking in three perpendicular directions, rotating ∼60° between streakings. Plates were allowed to dry for 3–5 min before disk application.

#### 2.5.1 Initial ESBL screening test

Initial screening for potential ESBL production was performed using third-generation cephalosporin disks: ceftazidime (30 μg), cefotaxime (30 μg), ceftriaxone (30 μg), and cefpodoxime (10 μg) (Oxoid Ltd., United Kingdom). Disks were aseptically placed on inoculated Mueller–Hinton Agar surfaces maintaining 25 mm center-to-center distance.

Plates were incubated aerobically at 37 °C for 16–18 h. Inhibition zone diameters were measured to the nearest millimeter using a calibrated digital Vernier caliper under reflected transmitted light. Isolates with inhibition zone diameters equal to or smaller than CLSI screening breakpoints for one or more indicator cephalosporins were classified as suspected ESBL producers and subjected to confirmatory testing.

#### 2.5.2 Phenotypic confirmatory test (Combination Disk Method)

Phenotypic confirmation was performed using the Combination Disk Test (CDT) per CLSI recommendations [31]. Two disk pairs were employed:

-Ceftazidime (30 μg), Ceftazidime + Clavulanic acid (30/10 μg), Cefotaxime (30 μg), Cefotaxime + Clavulanic acid (30/10 μg). (All disks supplied by Oxoid Ltd., United Kingdom.)

Disks were placed on freshly inoculated Mueller–Hinton Agar plates with 25–30 mm center-to-center distance to prevent inhibition zone interference.

Following 16–18 h incubation at 37 °C, inhibition zone diameters were measured independently by two experienced microbiologists. Discrepancies >2 mm were resolved by repeat testing. Per CLSI criteria, isolates demonstrating ≥5 mm increase in inhibition zone diameter around clavulanic acid-containing disks compared with corresponding cephalosporin disks alone were phenotypically confirmed as ESBL producers.

#### 2.5.3 Quality control

Quality control was performed daily using reference strains recommended by CLSI:

- *Escherichia coli* ATCC 25922 (ESBL-negative), *Klebsiella pneumoniae* ATCC 700603 (ESBL-positive). Measured inhibition zone diameters of quality-control strains were required to fall within CLSI acceptable ranges. Whenever results were outside limits, the assay was repeated after verification of inoculum density, media quality, incubation conditions, and disk potency.

#### 2.5.4 Quality assurance

Mueller–Hinton Agar plates were prepared per manufacturer instructions with sterility testing. Agar depth was maintained at 4 ± 0.5 mm, and final medium pH was 7.2–7.4 per CLSI.

Antimicrobial disks were stored at 2–8 °C in sealed desiccated containers and equilibrated to room temperature for ∼30 min before use. Each isolate was tested independently on three separate occasions, with only reproducible results included. Laboratory personnel performing measurements were blinded to isolate identity.

Phenotypically confirmed ESBL-producing isolates were selected for evaluation of antibacterial activity of biosynthesized AgNPs, MIC, MBC, and antibiofilm assays.

### 2.6 Antimicrobial susceptibility testing (Kirby–Bauer Disk Diffusion Method)

Antimicrobial susceptibility profiles of phenotypically confirmed ESBL-producing *E. coli* and *K. pneumonia* isolates were determined using the standardized Kirby–Bauer disk diffusion method per CLSI M100 (2024) to evaluate multidrug-resistant (MDR) characteristics before assessing AgNP antibacterial efficacy.^23^

#### 2.6.1 Preparation of bacterial inoculum

Fresh cultures were obtained by subculturing each isolate on Mueller–Hinton Agar (Oxoid Ltd., Basingstoke, United Kingdom) and incubating aerobically at 37 ± 1 °C for 18–24 h.

Three to five morphologically identical colonies were selected using a sterile inoculating loop and suspended in 5 mL of sterile physiological saline (0.85% NaCl). The suspension was adjusted to 0.5 McFarland turbidity (∼1.5 × 10^8^ CFU mL^−1^) using a DensiCHEK Plus densitometer (bioMérieux, Marcy-l'Étoile, France). Inocula were prepared immediately before testing and used within 15 min.

#### 2.6.2 Preparation of mueller–hinton agar plates

Commercial Mueller–Hinton Agar (Oxoid Ltd., United Kingdom) was prepared per manufacturer instructions using purified distilled water and sterilized by autoclaving at 121 °C for 15 min under 15 psi pressure. Approximately 25 mL of molten agar was poured into sterile 90-mm Petri dishes for uniform agar depth of 4.0 ± 0.5 mm per CLSI. Medium pH was verified after sterilization and maintained at 7.2–7.4 at room temperature. Prepared plates were inspected for contamination, dehydration, air bubbles, and surface irregularities before use. Plates were stored at 4 °C in sealed plastic bags and equilibrated to room temperature for ∼30 min before inoculation.

#### 2.6.3 Inoculation of test plates

A sterile cotton swab was immersed in standardized bacterial suspension and rotated to ensure complete saturation. Excess inoculum was removed by pressing against the tube wall.

The entire agar surface was inoculated uniformly by streaking in three different directions with ∼60° rotation after each streaking to produce a homogeneous lawn. The swab was passed around the plate edge to ensure complete inoculation. Inoculated plates were allowed to stand for 3–5 min (not >15 min) before disk application.

#### 2.6.4 Antibiotic disks

Commercial antibiotic disks (Oxoid Ltd., Basingstoke, United Kingdom) were selected per CLSI recommendations for Enterobacterales, including:

Ampicillin (10 μg), Amoxicillin-clavulanic acid (20/10 μg), Cefotaxime (30 μg), Ceftazidime (30 μg), Ceftriaxone (30 μg), Cefepime (30 μg), Gentamicin (10 μg), Amikacin (30 μg), Ciprofloxacin(5 μg), Levofloxacin (5 μg), Tetracycline (30 μg), Trimethoprim-sulfamethoxazole (1.25/23.75 μg), Meropenem (10 μg), Imipenem (10 μg), Fosfomycin (200 μg), Nitrofurantoin (300 μg). Disks were aseptically placed on inoculated agar surfaces with at least 25 mm center-to-center distance using sterile forceps or a calibrated disk dispenser.

#### 2.6.5 Incubation and measurement

Plates were inverted and incubated aerobically at 37 ± 1 °C for 16–18 h. Inhibition zone diameters were measured to the nearest millimeter using a calibrated digital Vernier caliper under reflected transmitted light. Results were interpreted per CLSI M100 (2024) breakpoints. Isolates were categorized as susceptible, intermediate, or resistant. MDR was defined as acquired non-susceptibility to at least one agent in three or more antimicrobial categories. *E. coli* ATCC 25922 was used as quality control.

#### 2.6.6 Quality control

Quality control was performed daily using *E. coli* ATCC 25922. Measured inhibition zones were required to fall within CLSI acceptable ranges. If outside limits, the assay was repeated after verification of all variables. All Mueller–Hinton Agar plates met CLSI specifications for pH, depth, and sterility. Disks were stored under proper conditions (Supplementary1). Each isolate was tested independently three times with reproducible results. Laboratory personnel were blinded to isolate identity. Antibiotic disks were stored at 2–8 °C in moisture-proof containers containing fresh desiccant and were brought to room temperature before application.

#### 2.6.7 Disk placement and incubation

Antibiotic disks were applied to the inoculated Mueller–Hinton Agar plates using a sterile automatic disk dispenser. A maximum of six antibiotic disks was placed on each 90-mm agar plate to avoid overlapping inhibition zones.

The minimum center-to-center distance between adjacent disks was maintained at 24 mm, and disks were gently pressed using sterile forceps to ensure complete contact with the agar surface.

The plates were inverted and incubated aerobically at 37 ± 1 °C for 16–18 h.

#### 2.6.8 Measurement and interpretation of inhibition zones

After incubation, inhibition zone diameters were measured in millimeters using a calibrated digital Vernier caliper under reflected light. Each inhibition zone was measured independently by two experienced microbiologists who were blinded to isolate identity. In cases where measurement discrepancies exceeded 2 mm, the assay was repeated. Susceptibility categories (Susceptible, Intermediate, and Resistant) were interpreted strictly according to the CLSI M100 (2024) breakpoint criteria.

#### 2.6.9 Definition of multidrug resistance

Multidrug-resistant (MDR) isolates were defined according to the international consensus criteria proposed by Magiorakos et al. (2012). An isolate was considered MDR when it exhibited acquired non-susceptibility to at least one antimicrobial agent in three or more antimicrobial classes[32]**.**

#### 2.6.10 Quality control

Quality-control testing was performed daily using the following ATCC reference strains:

• *Escherichia coli* ATCC 25922, *Klebsiella pneumoniae* ATCC 700603

Measured inhibition zone diameters for the quality-control strains were required to fall within the CLSI acceptable quality-control ranges before patient isolate results were accepted. Whenever quality-control values fell outside the recommended ranges, the entire testing procedure was repeated after verification of media quality, inoculum density, incubation conditions, and antibiotic disk potency.

#### 2.6.11 Experimental reproducibility

All susceptibility tests were performed independently in triplicate using freshly prepared bacterial inocula. Laboratory personnel responsible for measuring inhibition zones were blinded to the experimental grouping to minimize observer bias. Mean inhibition zone diameters ± standard deviation were calculated for all isolates. The antimicrobial susceptibility profiles obtained from the Kirby–Bauer assay served as the baseline for subsequent evaluation of the antibacterial activity of biosynthesized silver nanoparticles, determination of minimum inhibitory concentration (MIC), minimum bactericidal concentration (MBC), and antibiofilm activity against MDR ESBL-producing *E. coli* and *K. pneumoniae* isolates.

### 2.7 Determination of the minimum inhibitory concentration (MIC) of biosynthesized silver nanoparticles

The minimum inhibitory concentration (MIC) of biosynthesized silver nanoparticles (AgNPs) against clinical ESBL-producing *Escherichia coli* and *Klebsiella pneumoniae* isolates was determined using the broth microdilution method in accordance with the Clinical and Laboratory Standards Institute (CLSI) guideline M07 (12th Edition, 2024), with minor modifications appropriate for nanoparticle-based antimicrobial agents[33].

#### 2.7.1 Preparation of silver nanoparticle stock suspension

Freeze-dried biosynthesized AgNPs were weighed using a calibrated analytical balance (Sartorius Entris II, Sartorius AG, Göttingen, Germany) and suspended in sterile ultrapure deionized water to obtain a stock concentration of 2048 μg/mL**.**

To ensure complete nanoparticle dispersion and minimize aggregation, the suspension was vortexed for 1 min followed by ultrasonication in an ultrasonic bath (Branson Ultrasonics 2800, Emerson, Danbury, CT, USA) operating at 40 kHz and 100 W for 20 min. The freshly prepared suspension was used immediately after sonication.

The stock suspension was prepared under aseptic conditions inside a Class II biological safety cabinet to avoid microbial contamination.

#### 2.7.2 Preparation of bacterial inoculum

Fresh overnight cultures of each ESBL-producing isolate were grown on Mueller–Hinton Agar at 37 ± 1 °C for 18–24 h. Three to five morphologically identical colonies were transferred into sterile saline (0.85% NaCl), and turbidity was adjusted to 0.5 McFarland standard, corresponding to approximately 1.5 × 10^8^ CFU mL^−1^**,** using a DensiCHEK Plus densitometer (bioMérieux, France). The standardized suspension was subsequently diluted 1:300 in sterile cation-adjusted Mueller–Hinton Broth (CAMHB) to obtain a final bacterial concentration of approximately 5 × 10^5^ CFU mL^−1^**,** as recommended by CLSI for broth microdilution assays.

#### 2.7.3 Preparation of serial two-fold dilutions

MIC testing was performed in sterile, flat-bottom 96-well polystyrene microtiter plates (Corning Inc., Corning, NY, USA). A total volume of 100 μL of sterile CAMHB was dispensed into each well. Two-fold serial dilutions of AgNPs were prepared across the plate to obtain final nanoparticle concentrations of:

1024,512,256,128,64,32,16,8,4,2,1,and0.5μg/mL

Following preparation of serial dilutions, 100 μL of the standardized bacterial inoculum was added to each well, resulting in a final reaction volume of 200 μL per well.

#### 2.7.4 Experimental controls

To ensure assay validity, the following controls were included in every microplate:

• **Sterility control:** CAMHB only (without bacteria or nanoparticles).
• **Growth control:** CAMHB plus bacterial inoculum without AgNPs.
• **Nanoparticle control:** CAMHB containing AgNPs without bacteria to evaluate any background turbidity produced by nanoparticles.
• **Positive antimicrobial control:** CAMHB containing bacterial inoculum plus a reference antibiotic (Meropenem) at CLSI-recommended concentrations.
• **Medium control:** CAMHB alone to verify broth clarity.

Each isolate was tested in triplicate, and the complete experiment was independently repeated on three separate occasions using freshly prepared nanoparticle suspensions.

#### 2.7.5 Incubation conditions

Microplates were covered with sterile adhesive sealing films to minimize evaporation and incubated aerobically at 37 ± 1 °C for 18–20 h without shaking.

Following incubation, plates were visually inspected for bacterial growth under uniform illumination before spectrophotometric confirmation.

#### 2.7.6 Determination of MIC and MBC

The minimum inhibitory concentration (MIC) and minimum bactericidal concentration (MBC) of the biosynthesized silver nanoparticles (AgNPs) were determined using the broth microdilution method in sterile 96-well microplates following CLSI guidelines with minor modifications. AgNP suspensions were prepared at the desired test concentrations (6.25, 12.5, 25, 50, and 100 μg/mL) by serial dilution from the stock solution. After inoculation with standardized bacterial suspensions (approximately 5 × 10^5^ CFU mL^−1^), the plates were incubated at 37 °C for 18–24 h. The MIC was defined as the lowest AgNP concentration that completely inhibited visible bacterial growth, whereas the MBC was determined by subculturing aliquots from wells without visible growth onto Mueller–Hinton agar plates and identifying the lowest concentration that produced no bacterial colony after incubation.

#### 2.7.7 Determination of bactericidal activity

The relationship between MIC and MBC values was evaluated by calculating the MBC/MIC ratio for each isolate.

Interpretation was performed according to commonly accepted microbiological criteria:

• **MBC/MIC ≤ 4:** Bactericidal activity
• **MBC/MIC > 4:** Bacteriostatic activity

This classification allowed differentiation between complete bacterial killing and reversible inhibition of bacterial multiplication.

#### 2.7.8 Experimental controls

Each experimental run included the following controls:

• **Sterility control:** Mueller–Hinton Agar without bacterial inoculation.
• **Growth control:** Untreated bacterial suspension plated on Mueller–Hinton Agar.
• **Nanoparticle control:** AgNP suspension plated without bacteria to verify sterility of the nanoparticle preparation.
• **Positive antimicrobial control:** Meropenem-treated bacterial suspension.

All controls were processed simultaneously under identical laboratory conditions.

#### 2.7.9 Experimental reproducibility

Each bacterial isolate was analyzed in triplicate, and the entire experiment was independently repeated on three different occasions using freshly prepared AgNP suspensions.

Only reproducible MBC values obtained in all independent experiments were included in the final statistical analysis.

#### 2.7.10 Quality assurance

Fresh Mueller–Hinton Agar plates were prepared for every experimental run.

The sterility of culture media was verified by incubating uninoculated control plates at 37 °C for 48 h before use.

The viability of bacterial inocula was confirmed by determining colony-forming units (CFU) from untreated growth controls.

All culture manipulations were performed inside a certified Class II Biological Safety Cabinet under aseptic conditions to minimize environmental contamination.

Reference strains (*Escherichia coli* ATCC 25922 and *Klebsiella pneumoniae* ATCC 700603) were included in every assay to monitor inter-assay consistency and laboratory performance

The bactericidal efficacy of AgNPs was subsequently correlated with bacterial species, antimicrobial resistance profile, ESBL phenotype, and biofilm-forming ability to investigate possible associations between resistance characteristics and nanoparticle susceptibility.

The MBC data were interpreted together with MIC findings to provide a comprehensive evaluation of the antibacterial potential of biosynthesized silver nanoparticles against multidrug-resistant clinical isolates.

All experiments were performed using at least three independent biological replicates, with each measurement conducted in triplicate unless otherwise specified. Data are presented as mean ± standard deviation (SD). Statistical analyses were performed using GraphPad Prism (version XX, GraphPad Software, San Diego, CA, USA). Data normality was assessed using the Shapiro–Wilk test, while homogeneity of variance was evaluated using Levene's test. Comparisons between two groups were performed using an unpaired Student's *t*-test, whereas comparisons among multiple groups were analyzed using one-way analysis of variance (ANOVA) followed by Tukey's multiple comparison post hoc test. When the assumptions of normality or homogeneity were not satisfied, appropriate non-parametric tests were considered. Statistical significance was defined as *P* < 0.05. Where appropriate, effect sizes, and 95% confidence intervals are reported to quantitative Biofilm.

### 2.8 Quantitative biofilm formation assay

The antibiofilm activity of biosynthesized silver nanoparticles (AgNPs) against ESBL-producing *Escherichia coli* and *Klebsiella pneumoniae* was evaluated using the crystal violet microtiter plate assay with minor modifications. Briefly, overnight bacterial cultures were adjusted to **0.5 McFarland** (approximately **1.5 × 10**^**8**^ **CFU mL**^**−1**^) and diluted 1:100 in tryptic soy broth (TSB) supplemented with **1% glucose**.

Aliquots (100 μL) of the bacterial suspension were added to sterile 96-well polystyrene microplates containing equal volumes of AgNP suspensions at final concentrations of **12.5, 25, 50, 100, and 200** μ**g/mL**. Wells containing bacteria without AgNPs served as the positive control, whereas wells containing sterile medium alone served as the negative control. After incubation at 37 °C for 24 h, planktonic cells were removed, and the wells were gently washed three times with sterile phosphate-buffered saline (PBS) to eliminate non-adherent bacteria.

The attached biofilms were fixed with methanol for **15 min**, stained with **0.1% (*w*/*v*) crystal violet** for **15 min**, and washed thoroughly with distilled water to remove excess stain. The bound crystal violet was then solubilized with **95% ethanol**, and the absorbance was measured at **570 nm** using a microplate reader.

Biofilm inhibition (%) was calculated relative to the untreated control using the following equation:

Biofilm inhibition (%) = [(ODcontrol − ODtreated) / ODcontrol] × 100.

All experiments were performed in triplicate (*n* = 3), and the results are expressed as mean ± SD**.** Statistical significance was determined using one-way ANOVA followed by Tukey's multiple comparison test**,** with *P* < 0.05 considered statistically significant.

### 2.9 Evaluation of the antibiofilm activity of biosynthesized silver nanoparticles

The antibiofilm activity of biosynthesized silver nanoparticles (AgNPs) against ESBL-producing *Escherichia coli* and *Klebsiella pneumoniae* was evaluated using the crystal violet microtiter plate assay. Only isolates previously identified as moderate or strong biofilm producers were included. Overnight bacterial cultures were adjusted to approximately 1 × 10^6^ CFU mL^−1^, and 200 μL aliquots were inoculated into sterile 96-well polystyrene microplates. After incubation at 37 °C for 24 h, planktonic cells were removed, and the wells were gently washed three times with sterile phosphate-buffered saline (PBS) to obtain mature biofilms.

Freshly prepared AgNP suspensions were added to the preformed biofilms at final concentrations of 12.5, 25, 50, 100, and 200 μg/mL for the quantitative antibiofilm assay. For determination of the minimum biofilm eradication concentration (MBEC), additional AgNP concentrations ranging up to 3200 μg/mL were evaluated to identify the lowest concentration that completely eradicated established biofilms. Untreated biofilms, sterile medium, AgNP suspensions without bacteria, and meropenem-treated biofilms served as the control groups. Following an additional 24 h of incubation, the wells were washed, fixed with methanol, stained with 0.1% crystal violet, and the bound dye was solubilized with 33% acetic acid. Biofilm biomass was quantified by measuring the absorbance at 570 nm using a microplate reader. The percentage of biofilm inhibition was calculated using the following equation:

$$\text{Biofilm inhibition}\;\left(\%\right)=\left[\left(\mathrm{OD}\_\text{control}-\mathrm{OD}\_\text{treated}\right)/\mathrm{OD}\_\text{control}\right]\times 100$$

The MBEC was defined as the lowest AgNP concentration that completely prevented bacterial regrowth following recovery of treated biofilms on Mueller–Hinton agar. Representative untreated and AgNP-treated biofilms were further examined by field emission scanning electron microscopy (FESEM) to evaluate structural alterations in the biofilm architecture and bacterial morphology. All experiments were performed in three independent experiments, each with eight technical replicates, and the results are expressed as mean ± SD. Statistical analysis was performed using one-way ANOVA followed by Tukey's multiple comparison test, with *P* < 0.05 considered statistically significant.

#### 2.9.1 Data analysis

Results were expressed as:

• Mean optical density (OD570 ± SD)
• Percentage biofilm inhibition
• MBEC values (μg/mL)

Comparisons were performed separately for *Escherichia coli* and *Klebsiella pneumoniae*.

The antibiofilm activity of AgNPs was subsequently correlated with:

• MIC values
• MBC values
• Initial biofilm-forming ability
• ESBL phenotype
• Antimicrobial resistance profile

to investigate factors influencing nanoparticle-mediated biofilm eradication.

### 2.10 Time–kill kinetic assay

The bactericidal kinetics of biosynthesized silver nanoparticles (AgNPs) against ESBL-producing *Escherichia coli* and *Klebsiella pneumoniae* were evaluated using a standardized time–kill assay to determine the rate and extent of bacterial killing over time. The assay was performed according to CLSI recommendations and internationally accepted methodologies for antimicrobial pharmacodynamic studies, with modifications appropriate for nanoparticle-based antimicrobial agents.

### 2.11 Selection of bacterial isolates

Representative clinical isolates exhibiting multidrug resistance, confirmed ESBL production, and moderate or strong biofilm-forming capacity were selected for kinetic analysis. In addition, the reference strains *Escherichia coli* ATCC 25922 and *Klebsiella pneumoniae* ATCC 700603 were included as quality-control organisms.

Fresh overnight cultures grown on Mueller–Hinton Agar were suspended in sterile saline and adjusted to 0.5 McFarland standard (approximately 1.5 × 10^8^ CFU mL^−1^). The suspension was diluted in cation-adjusted Mueller–Hinton Broth (CAMHB) to obtain a final bacterial concentration of approximately 5 × 10^5^ CFU mL^−1^.

#### 2.11.1 Preparation of AgNP working solutions

Fresh AgNP suspensions were prepared immediately before each experiment and dispersed by ultrasonication (40 kHz, 100 W) for 15 min.

The nanoparticles were tested at four concentrations:

• 0.5 × MIC
• 1 × MIC
• 2 × MIC
• 4 × MIC

Each concentration was prepared in sterile CAMHB under aseptic conditions.

### 2.12 Experimental design

Sterile 50-mL polypropylene centrifuge tubes containing 10 mL of CAMHB were inoculated with the standardized bacterial suspension and mixed with AgNPs to obtain the desired final concentrations.

Control groups included:

• Growth control (bacteria without AgNPs)
• Sterility control (CAMHB only)
• AgNP control (AgNPs without bacteria)
• Positive antimicrobial control (Meropenem at CLSI-recommended concentration)

All tubes were incubated at **37 ± 1 °C** with continuous shaking at **150 rpm** to maintain homogeneous nanoparticle dispersion and adequate aeration.

### 2.13 Sampling schedule

Aliquots (**100** μ**L**) were collected aseptically at the following time points:

• 0 h
• 2 h
• 4 h
• 6 h
• 8 h
• 12 h
• 24 h

Immediately after sampling, serial ten-fold dilutions were prepared in sterile phosphate-buffered saline (PBS).

#### 2.13.1 Viable bacterial enumeration

Appropriate dilutions were spread onto Mueller–Hinton Agar plates using the spread plate technique.

The inoculated plates were incubated aerobically at **37 °C for 24 h**, after which viable colonies were counted using a calibrated digital colony counter.

Results were expressed as log**₁₀ CFU mL**^**−1**^ for each sampling time.

#### 2.13.2 Interpretation of bactericidal activity

A reduction of **≥3 log₁₀ CFU mL**^**−1**^ relative to the initial bacterial inoculum was interpreted as **bactericidal activity**, whereas reductions below this threshold were considered bacteriostatic.

Complete absence of bacterial growth after incubation was interpreted as total bacterial eradication.

#### 2.13.3 Experimental reproducibility

Each isolate was tested in **triplicate**, and the entire experiment was independently repeated **three times**.

Mean bacterial counts and standard deviations were calculated for every sampling point.

#### 2.13.4 Data analysis

Time–kill curves were constructed by plotting **log₁₀ CFU mL**^**−1**^ against incubation time.

The rate of bacterial killing at different AgNP concentrations was compared between *Escherichia coli* and *Klebsiella pneumoniae*.

The kinetic results were interpreted together with MIC, MBC, and antibiofilm findings to provide a comprehensive evaluation of the antibacterial activity of biosynthesized AgNPs.

#### 2.13.5 Cytocompatibility assessment of *Saussurea costus*-mediated AgNPs

The cytocompatibility of the biosynthesized *Saussurea costus*-mediated silver nanoparticles (AgNPs) was evaluated using human dermal fibroblast adult (HDFa) cells by the MTT colorimetric assay. HDFa cells were cultured in Dulbecco's Modified Eagle Medium (DMEM; Gibco, USA) supplemented with 10% fetal bovine serum (FBS), 1% penicillin–streptomycin solution, and 1% l-glutamine under standard cell culture conditions (37 °C in a humidified atmosphere containing 5% CO₂).

Cells were seeded into sterile 96-well tissue culture plates at an appropriate density and allowed to adhere for 24 h. Subsequently, the culture medium was replaced with fresh medium containing serial concentrations of biosynthesized AgNPs (64, 128, 256, 512, and 1024 μg/mL). Untreated cells cultured in nanoparticle-free medium served as the negative control. Following 24 h of incubation, the treatment medium was removed and each well was incubated with MTT solution (0.5 mg/mL) for 3–4 h at 37 °C to allow the formation of intracellular formazan crystals by metabolically active cells.

After incubation, the supernatant was carefully discarded and the formazan crystals were dissolved in dimethyl sulfoxide (DMSO). The absorbance of each well was measured at 570 nm using a microplate reader. Cell viability was calculated as the percentage of absorbance in treated cells relative to untreated control cells according to the following equation:

$$\text{Cell viability}\;\left(\%\right)=\left(\text{Absorbance of treated cells}/\text{Absorbance of control cells}\right)\times 100$$

Dose–response curves were constructed to determine the half-maximal inhibitory concentration (IC₅₀) of the synthesized AgNPs toward HDFa cells. According to ISO 10993-5 guidelines, cell viability values ≥70% were considered indicative of acceptable in vitro cytocompatibility.

### 2.14 Statistical analysis

All experiments were performed in triplicate (*n* = 3) unless otherwise stated, and the results are presented as mean ± standard deviation (SD)**.** Statistical analyses were performed using IBM SPSS Statistics version XX (IBM Corp., Armonk, NY, USA)**.**

Differences in antimicrobial susceptibility profiles between *Escherichia coli* and *Klebsiella pneumoniae* were analyzed using the Chi-square test or Fisher's exact test, as appropriate. To control the family-wise error rate resulting from multiple comparisons across the 12 tested antibiotics, Bonferroni correction was applied, and statistical significance was defined as an adjusted *P* < 0.0042 (0.05/12).

For quantitative data, including inhibition zone diameters, minimum inhibitory concentration (MIC), minimum bactericidal concentration (MBC), biofilm inhibition, and cytotoxicity assays, statistical differences among experimental groups were evaluated using one-way analysis of variance (ANOVA) followed by Tukey's multiple comparison test. Comparisons between *E. coli* and *K. pneumoniae* at the same treatment concentration were performed using the independent**-**samples Student's *t*-test. A value of *P* < 0.05 was considered statistically significant for these analyses.

## 3 Results

### 3.1 Isolation and identification of clinical isolates

A total of 229 non-duplicate clinical isolates were recovered from various clinical specimens collected from patients attending major hospitals in Erbil City, Kurdistan Region, Iraq. Based on conventional microbiological identification, the isolates comprised 129 ***Escherichia coli*** and 100 ***Klebsiella pneumoniae***. Preliminary identification was established according to colony morphology, Gram staining characteristics, and standard biochemical tests. The distribution of the collected isolates and the representative isolates included in subsequent molecular and antimicrobial investigations are summarized in Table 1.

**Table 1:** Distribution of clinical isolates and representative ESBL-producing *Escherichia coli* and *Klebsiella pneumoniae* isolates included in the study.

Characteristic	*Escherichia coli*	*Klebsiella pneumoniae*	Total
Clinical isolates recovered	129	100	229
Representative isolates selected for molecular characterization	10	10	20
Representative isolates subjected to 16S rRNA gene sequencing	10	10	20
Representative isolates evaluated for AgNP susceptibility (MIC and MBC)	10	10	20
Representative isolates included in biofilm and antibiofilm assays	10	10	20

**Note:** Data are presented as the number of isolates. AgNPs: biosynthesized silver nanoparticles.

Following phenotypic screening for extended-spectrum β-lactamase (ESBL) production, representative multidrug-resistant ESBL-producing isolates were selected for molecular characterization. A total of 20 representative isolates, including 10 ***E. coli*** and 10 ***K. pneumoniae***, were subjected to amplification of the bacterial 16S rRNA gene using universal primers.

**Fig. 1:**
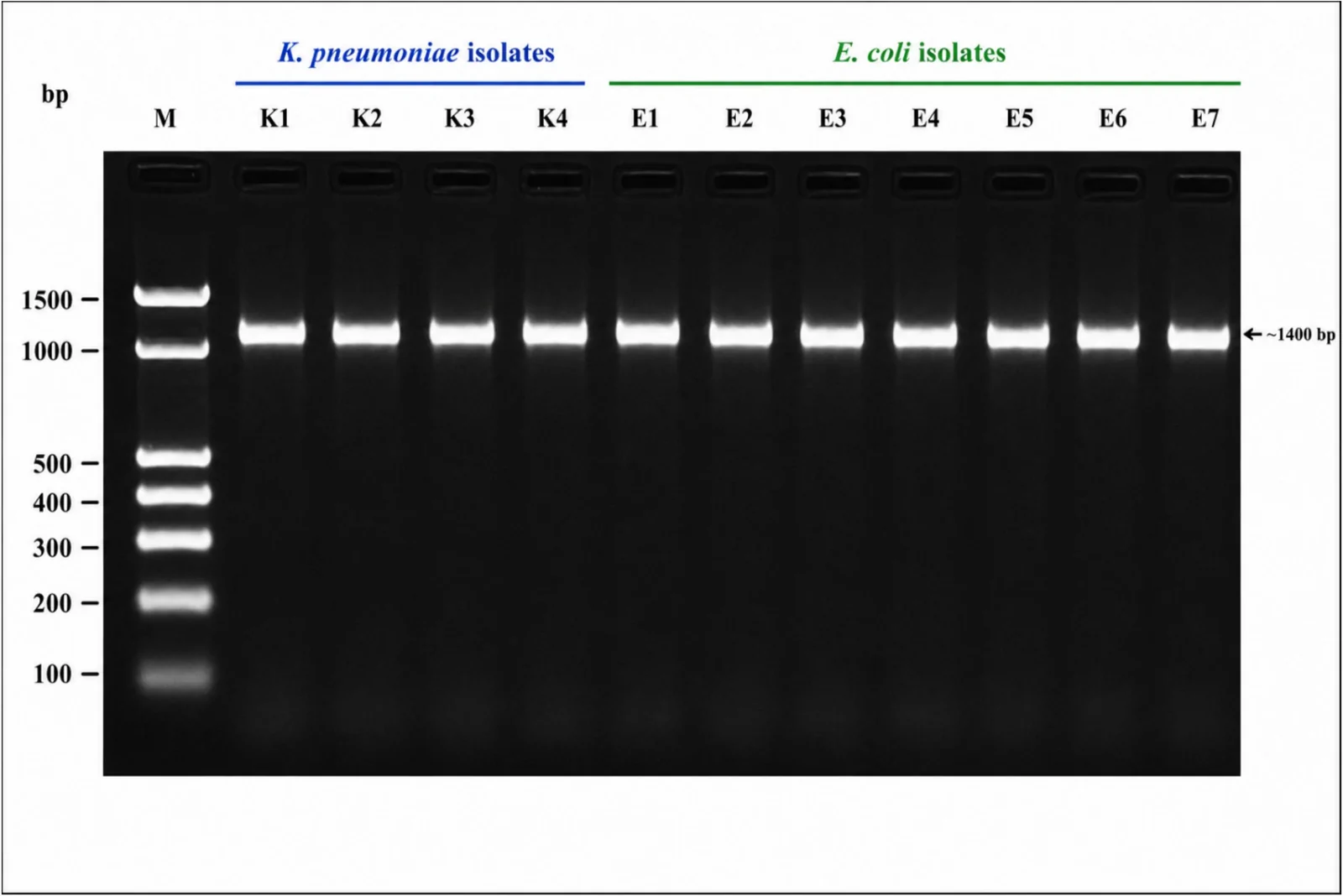
Agarose gel electrophoresis of PCR-amplified 16S rRNA gene fragments from representative ESBL-producing *Klebsiella pneumoniae* and *Escherichia coli* clinical isolates. Lane M represents the 100-bp DNA ladder used as the molecular size marker. Lanes K1–K4 correspond to representative *K. pneumoniae* isolates, whereas lanes E1–E7 represent representative *E. coli* isolates. A distinct amplification product of approximately 1400 bp was observed in all examined isolates without detectable nonspecific amplification, confirming successful PCR amplification of the bacterial 16S rRNA gene prior to sequencing and molecular identification.

PCR amplification successfully produced the expected amplicon of approximately 1400 bp in all representative isolates. Agarose gel electrophoresis demonstrated a single, well-defined amplification band of the expected size without detectable nonspecific products or primer-dimer formation, confirming successful amplification of the target gene. A representative electrophoretic profile of the amplified 16S rRNA fragments is presented in Fig. 1.

The amplified PCR products were purified and sequenced, and the obtained nucleotide sequences were analyzed using the BLASTn algorithm against the National Center for Biotechnology Information (NCBI) nucleotide database. Sequence alignment confirmed the molecular identity of all representative isolates, showing complete correspondence with reference sequences of *Escherichia coli* and *Klebsiella pneumoniae*. Representative nucleotide sequences generated in the present study were subsequently submitted to the GenBank database and assigned accession numbers, as summarized in Table 1.

**Fig. 2:**
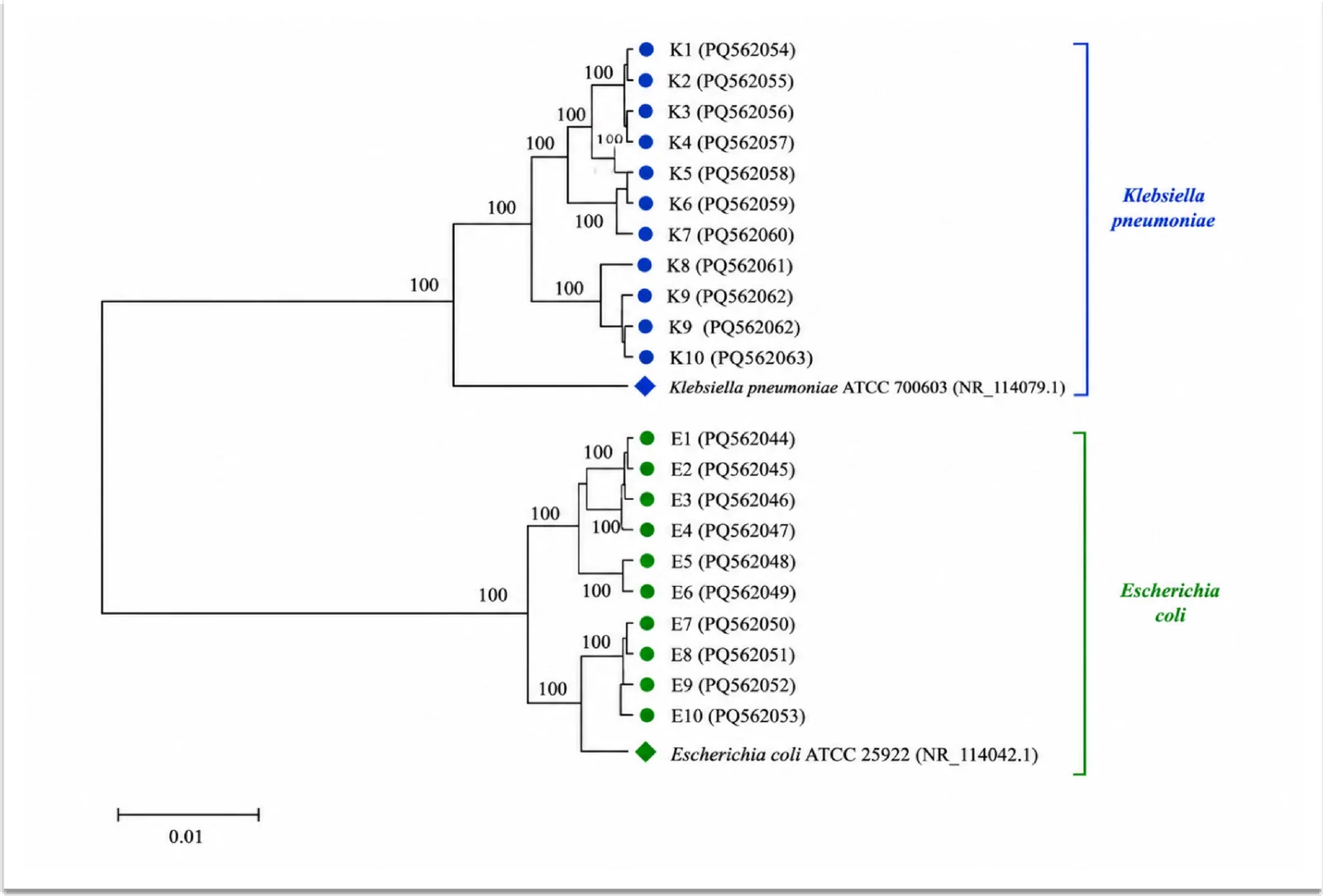
Maximum-likelihood phylogenetic tree based on partial 16S rRNA gene sequences of representative ESBL-producing *Klebsiella pneumoniae* (K1−K10) and *Escherichia coli* (E1–E10) clinical isolates. The phylogenetic tree was constructed using the Maximum-Likelihood method with 1000 bootstrap replicates. Representative isolates clustered with their corresponding reference strains retrieved from the NCBI GenBank database, confirming their molecular identification. Bootstrap values are indicated at the nodes, and the scale bar represents the number of nucleotide substitutions per site.

Phylogenetic relationships inferred from the partial 16S rRNA gene sequences demonstrated that all representative *E. coli* and *K. pneumoniae* isolates clustered within their respective species-specific lineages together with corresponding reference strains retrieved from the GenBank database. The reconstructed phylogenetic tree showed clear separation between the two bacterial species and confirmed the taxonomic placement of all analyzed isolates (Fig. 2).

The molecularly confirmed representative isolates were subsequently used for antimicrobial susceptibility testing, determination of ESBL-associated genes, evaluation of the antibacterial and antibiofilm activities of biosynthesized silver nanoparticles, minimum inhibitory concentration (MIC) and minimum bactericidal concentration (MBC) assays, as well as time-kill kinetic analyses.

### 3.2 Phenotypic detection of ESBL-producing isolates and antimicrobial susceptibility profiles

All recovered *Escherichia coli* and *Klebsiella pneumoniae* isolates were screened for extended-spectrum β-lactamase (ESBL) production using the combination disk diffusion method in accordance with CLSI recommendations. Phenotypically confirmed ESBL-producing isolates subsequently underwent antimicrobial susceptibility testing against a panel of β-lactam and non-β-lactam antibiotics.

The antimicrobial susceptibility profiles of the clinical isolates are summarized in Table 2. Overall, both bacterial species exhibited extensive resistance to commonly prescribed β-lactam antibiotics, including ampicillin, cefotaxime, ceftazidime, cefepime, and aztreonam. High resistance rates were also observed against fluoroquinolones and trimethoprim/sulfamethoxazole, whereas aminoglycosides, particularly amikacin, retained comparatively greater antimicrobial activity. Carbapenems (imipenem and meropenem) demonstrated the highest in vitro activity against the majority of the examined isolates.

**Table 2:** Comparison of antimicrobial susceptibility profiles of ESBL-producing *Escherichia coli* and *Klebsiella pneumoniae* clinical isolates. Statistical significance was evaluated using the Chi-square or Fisher's exact test with Bonferroni correction for multiple comparisons.

Antibiotic	Original P	Bonferroni-adjusted significance
Ampicillin	**0.004**	**Significant** ✅
Cefotaxime	0.332	Not significant
Ceftazidime	0.280	Not significant
Cefepime	0.357	Not significant
Aztreonam	0.214	Not significant
Gentamicin	**0.040**	Not significant ❌
Amikacin	0.088	Not significant
Ciprofloxacin	0.162	Not significant
Levofloxacin	0.149	Not significant
Trimethoprim/Sulfamethoxazole	**0.020**	Not significant ❌
Meropenem	0.604	Not significant
Imipenem	1.000	Not significant

Note: Data are presented as number (%) of isolates. Differences between *Escherichia coli* and *Klebsiella pneumoniae* were analyzed using the Chi-square test or Fisher's exact test, as appropriate. To account for multiple comparisons across antibiotics, P values were adjusted using the Bonferroni correction. Statistical significance was defined as adjusted *P* < 0.0042. Bold values indicate statistically significant differences after Bonferroni correction.

Comparison of the antimicrobial susceptibility profiles between *E. coli* and *K. pneumoniae* was performed using the Chi-square test or Fisher's exact test, as appropriate. To account for multiple comparisons across the 12 tested antibiotics, Bonferroni correction was applied. Following adjustment, a statistically significant interspecies difference was observed only for ampicillin susceptibility (*P* = 0.004), whereas no significant differences were detected for the remaining antimicrobial agents after correction for multiple testing (Table 2).

**Fig. 3:**
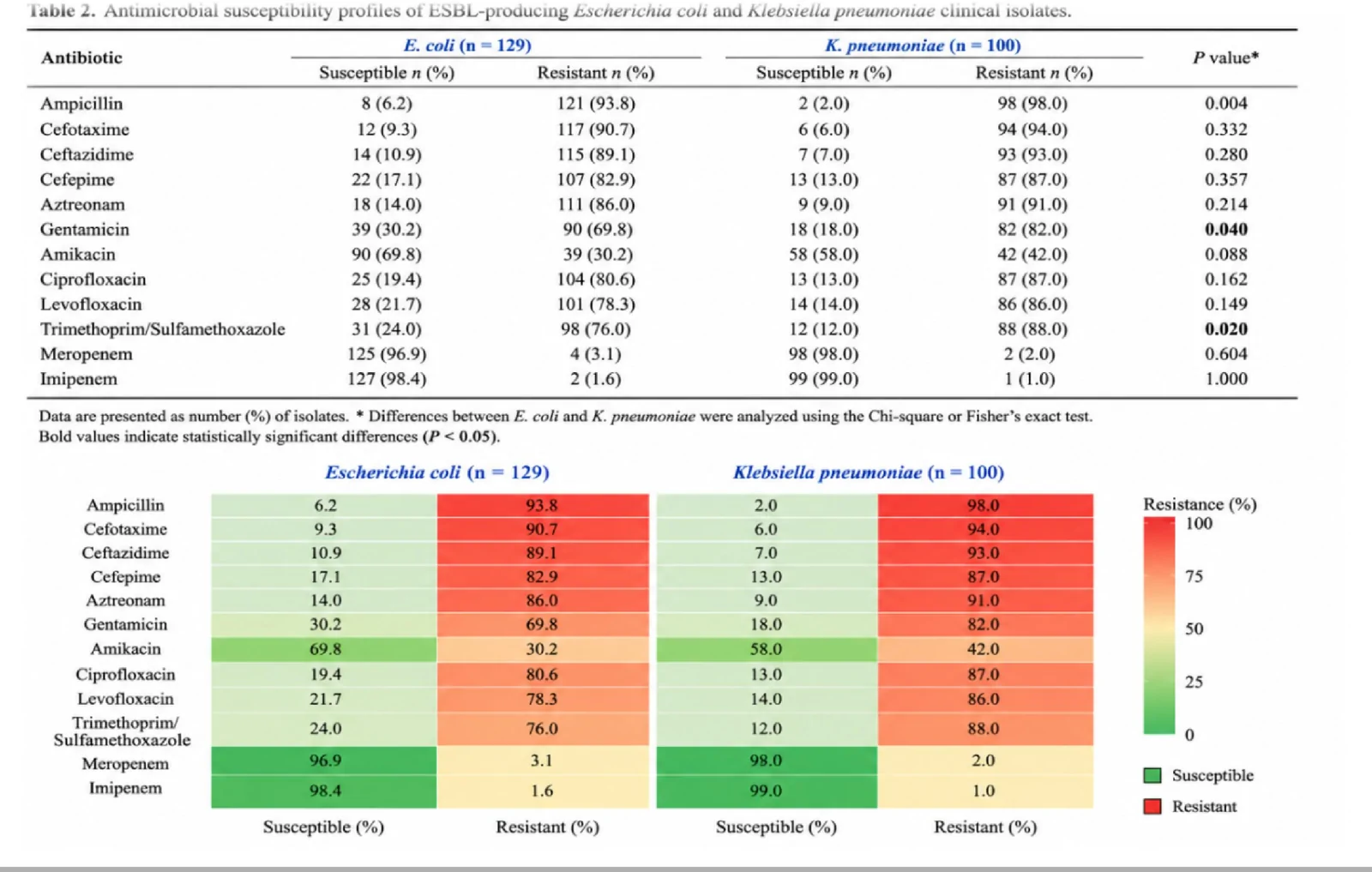
Heatmap illustrating the antimicrobial susceptibility profiles of ESBL-producing *Escherichia coli* (*n* = 129) and *Klebsiella pneumoniae* (*n* = 100) clinical isolates against the tested antimicrobial agents. Color intensity represents the percentage of susceptible and resistant isolates for each antibiotic, providing a comparative overview of resistance patterns between the two bacterial species.

To facilitate visualization of the resistance patterns, a heatmap was generated based on the percentage of resistant isolates for each antimicrobial agent (Fig. 3). The heatmap illustrates the overall distribution of antimicrobial resistance across both bacterial species and highlights the predominance of resistance to β-lactam antibiotics, while demonstrating the preserved activity of carbapenems against most isolates. The graphical representation also provides a direct comparison of resistance profiles between *E. coli* and *K. pneumoniae*, emphasizing species-specific variations in susceptibility patterns.

The antimicrobial susceptibility results confirmed the multidrug-resistant phenotype of the representative ESBL-producing isolates selected for subsequent evaluation of the antibacterial efficacy of biosynthesized silver nanoparticles.

### 3.3 Physicochemical characterization of biosynthesized silver nanoparticles

#### 3.3.1 Visual observation and UV–visible spectroscopy

The biosynthesis of silver nanoparticles (AgNPs) using the aqueous root extract of *Saussurea costus* was initially confirmed by a distinct visual color change during the reaction process. Upon mixing the plant extract with silver nitrate solution, the reaction mixture gradually changed from a light greenish-brown color to a dark brown suspension within approximately 3 h, whereas the control solutions containing either AgNO₃ alone or plant extract without silver ions showed no visible color change. This characteristic color transition indicates the reduction of Ag^+^ ions into metallic Ag^0^ nanoparticles and is attributed to the excitation of localized surface plasmon resonance (LSPR) on the nanoparticle surface.

**Fig. 4:**
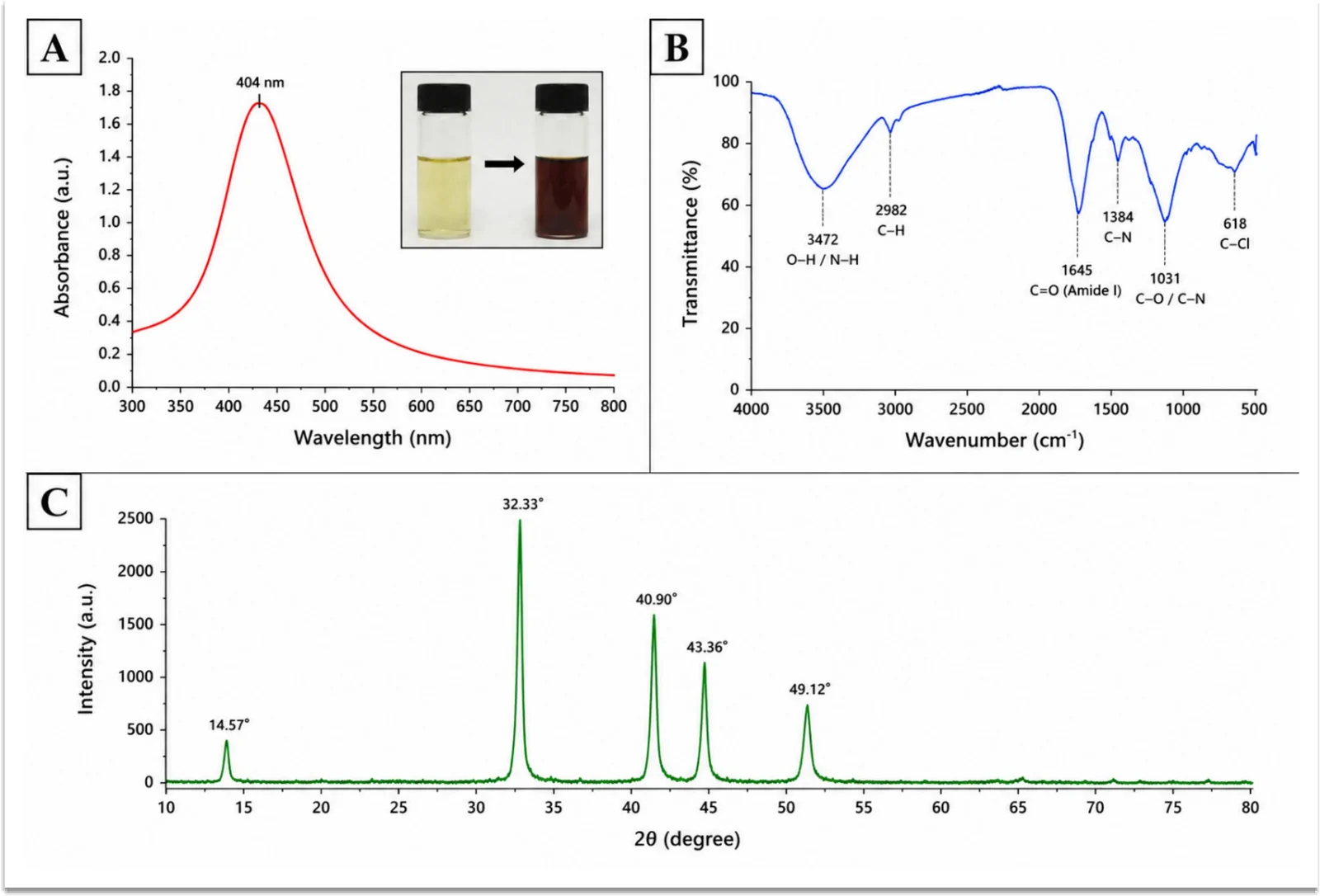
Physicochemical characterization of biosynthesized silver nanoparticles (AgNPs) synthesized using *Saussurea costus* root extract. (A) UV–Visible absorption spectrum showing the characteristic surface plasmon resonance (SPR) peak at approximately 404 nm together with the visible color change accompanying nanoparticle formation. (B) FTIR spectra of *S. costus* root extract and biosynthesized AgNPs demonstrating the principal functional groups involved in the reduction of Ag^+^ ions and stabilization of the synthesized nanoparticles. (C) X-ray diffraction (XRD) pattern confirming the crystalline nature of the AgNPs and the successful formation of metallic silver nanoparticles.

The UV–Visible absorption spectrum further confirmed nanoparticle formation by exhibiting a well-defined surface plasmon resonance (SPR) absorption band centered at 404 nm (Fig. 4A). The appearance of a single, narrow absorption peak is consistent with the successful biosynthesis of silver nanoparticles exhibiting relatively uniform size distribution. Time-dependent spectral monitoring demonstrated a gradual increase in absorbance intensity throughout the reaction period without the appearance of additional absorption bands, indicating continuous nanoparticle formation and progressive reduction of silver ions. No comparable absorption peak was detected in the corresponding control samples, confirming that the phytochemical constituents of *S. costus* root extract were responsible for both the reduction of Ag^+^ ions and the stabilization of the synthesized nanoparticles.

The observed SPR peak at 404 nm agrees well with the characteristic optical behavior of biologically synthesized silver nanoparticles and provides spectroscopic evidence supporting the successful green synthesis of stable AgNPs using *S. costus* root extract.

#### 3.3.2 Fourier transform infrared (FTIR) analysis

Fourier transform infrared (FTIR) spectroscopy was performed to identify the functional groups present in the *Saussurea costus* root extract and to elucidate their involvement in the reduction and stabilization of the biosynthesized silver nanoparticles (Fig. 4B). Comparison of the spectra obtained from the crude extract and purified AgNPs revealed similar absorption profiles with slight shifts in peak positions, indicating the adsorption of plant-derived biomolecules onto the nanoparticle surface.

The broad absorption band observed at **3408–3422 cm**^**−1**^ was assigned to O—H stretching vibrations of phenolic compounds and flavonoids. Peaks detected within **2924–2972 cm**^**−1**^ corresponded to aliphatic C—H stretching vibrations. Characteristic absorption bands appearing at **1633–1645 cm**^**−1**^ were attributed to amide I (C=O) stretching of proteins and other carbonyl-containing biomolecules, whereas bands around **1384–1407 cm**^**−1**^ were associated with aromatic C=C and C—N stretching vibrations. Strong absorptions within **1050–1261 cm**^**−1**^ were assigned to C—O stretching of alcohols, ethers, and phenolic compounds, while weaker bands below **1000 cm**^**−1**^ corresponded to out-of-plane bending vibrations of aromatic C—H groups.

Minor shifts in the positions and intensities of these absorption bands following nanoparticle synthesis indicate interactions between the functional groups of *S. costus* phytochemicals and the AgNP surface. These findings suggest that naturally occurring phenolics, flavonoids, proteins, and other oxygen-containing biomolecules served both as reducing agents for the conversion of Ag^+^ ions into metallic Ag^0^ nanoparticles and as capping agents responsible for nanoparticle stabilization.

#### 3.3.3 X-ray diffraction (XRD) analysis

The crystalline structure and phase purity of the biosynthesized silver nanoparticles (AgNPs) were investigated by X-ray diffraction (XRD), and the diffraction pattern is presented in Fig. 4C. The XRD spectrum exhibited four characteristic diffraction peaks at 2θ = 38.1°, 44.3°, 64.4°, and 77.5°, corresponding to the **(**111**), (**200**), (**220**),** and **(**311**)** crystallographic planes, respectively, of face-centered cubic (FCC) metallic silver (Ag^0^). These diffraction peaks are in excellent agreement with the standard diffraction data for crystalline silver (JCPDS Card No. 04–0783), confirming the successful formation of metallic silver nanoparticles through the green synthesis approach.

As shown in Fig. 4C, the diffraction peak corresponding to the **(**111**)** plane exhibited the highest intensity, indicating the preferential orientation and high crystallinity of the synthesized AgNPs. Furthermore, no additional prominent diffraction peaks corresponding to crystalline silver oxide or other impurity phases were detected, suggesting the high phase purity of the synthesized nanoparticles. The observed peak broadening is consistent with the nanoscale crystallite size of the AgNPs. Overall, the XRD results confirmed the successful biosynthesis of highly crystalline face-centered cubic silver nanoparticles using *Saussurea costus* root extract. These findings are consistent with the UV–Vis, FTIR, FESEM, DLS, and zeta potential analyses, collectively demonstrating the successful synthesis and physicochemical characterization of the AgNPs.

#### 3.3.4 Field emission scanning Electron microscopy (FESEM) analysis

**Fig. 5:**
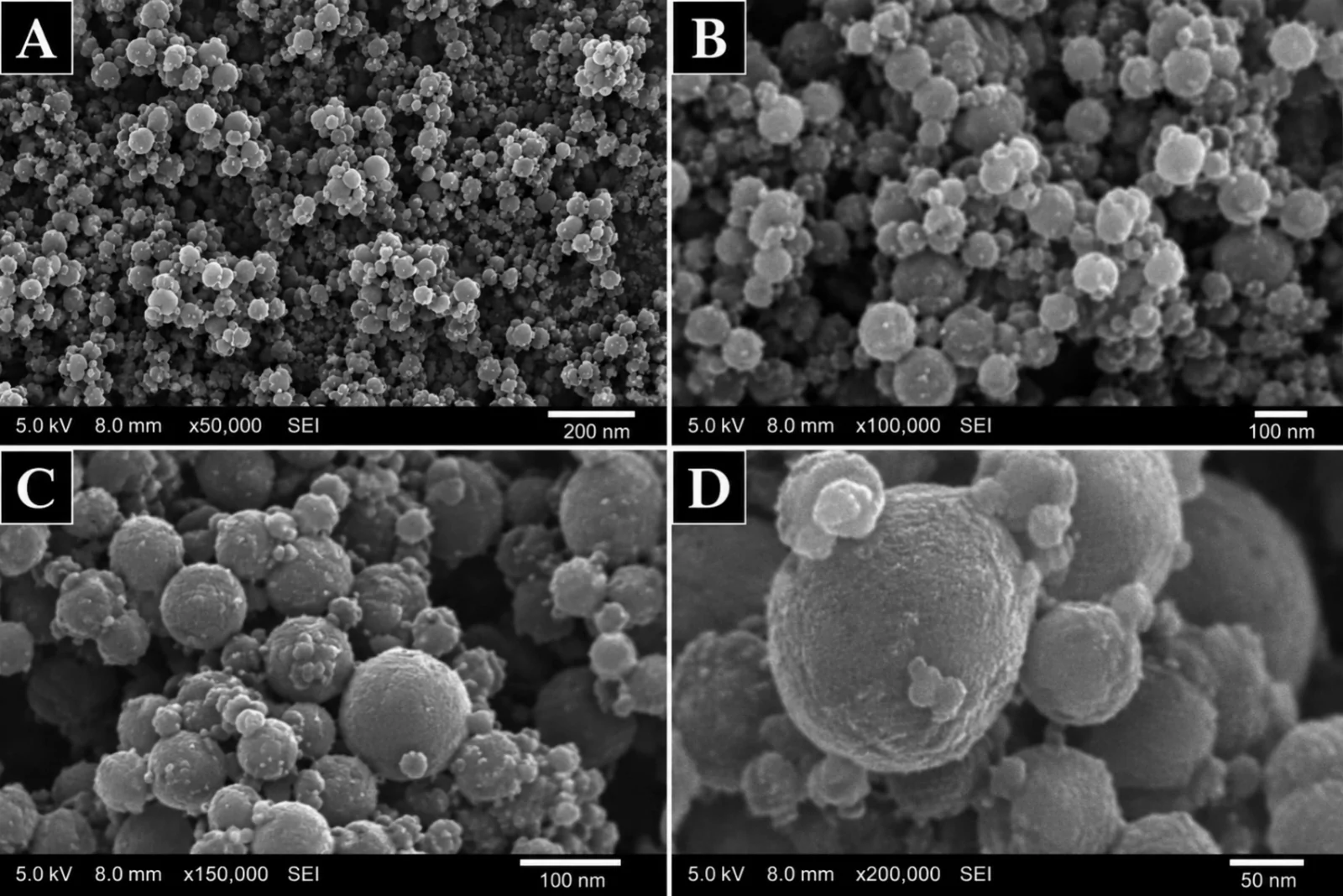
Field emission scanning electron microscopy (FESEM) characterization of biosynthesized silver nanoparticles (AgNPs) synthesized using *Saussurea costus* root extract. (A) Low-magnification FESEM image showing the overall distribution of the synthesized nanoparticles. (B) Higher-magnification micrograph illustrating particle morphology and dispersion. (C) FESEM image demonstrating nanoscale particle dimensions and surface characteristics. (D) High-magnification micrograph highlighting the predominantly spherical morphology with limited aggregation, indicating successful green synthesis of relatively uniform AgNPs. (For interpretation of the references to color in this figure legend, the reader is referred to the web version of this article.)

Field emission scanning electron microscopy (FESEM) images (Fig. 5A) revealed that the biosynthesized silver nanoparticles (AgNPs) were predominantly spherical with a relatively uniform distribution and only limited aggregation. The nanoparticles exhibited well-defined morphologies with smooth surfaces, indicating the successful formation of AgNPs through the green synthesis process. The observed particle morphology was consistent with the crystalline nature confirmed by XRD analysis.

Although FESEM provided valuable information regarding particle morphology and surface characteristics, the particle size distribution was determined more accurately by transmission electron microscopy (TEM) because of its higher spatial resolution. The FESEM observations were in good agreement with the TEM analysis, confirming the formation of nanosized silver nanoparticles with relatively uniform morphology.

#### 3.3.5 Energy-dispersive X-ray spectroscopy (EDX) analysis

The elemental composition of the biosynthesized silver nanoparticles was investigated using energy-dispersive X-ray spectroscopy (EDX), and the corresponding spectrum is presented in Fig. 5B. The analysis confirmed the successful formation of silver nanoparticles by revealing silver as the predominant element in the sample.

Quantitative elemental analysis demonstrated that silver constituted **67.99%** of the detected elements, while oxygen and sodium accounted for **26.62%** and **5.39%**, respectively. The presence of oxygen is attributed to oxygen-containing phytochemicals adsorbed onto the nanoparticle surface, whereas the minor sodium signal most likely originated from naturally occurring inorganic constituents of the plant extract or residual reaction components.

The predominance of elemental silver together with the relatively low abundance of other elements indicates the successful synthesis of AgNPs with high elemental purity. Furthermore, the detection of oxygen-containing components supports the FTIR findings, suggesting that phytochemical compounds from *Saussurea costus* root extract remained associated with the nanoparticle surface and contributed to their stabilization.

#### 3.3.6 Transmission Electron microscopy (TEM) analysis

Transmission electron microscopy (TEM) was employed to further characterize the morphology and particle size of the biosynthesized silver nanoparticles, and the representative micrographs are presented in Fig. 5C/D. The TEM images revealed that the synthesized AgNPs were predominantly spherical and well dispersed, with only limited aggregation observed among neighboring particles.

Particle size analysis demonstrated that the nanoparticles possessed nanoscale dimensions, with diameters ranging from **22.04 to 128.43 nm**. The average core particle size was estimated to be approximately **22 nm**, confirming the successful synthesis of nanocrystalline silver particles. The relatively narrow distribution of most particles indicates that the phytochemical constituents of *Saussurea costus* root extract effectively controlled nanoparticle nucleation and growth during the biosynthesis process.

The particle morphology observed by TEM was consistent with the FESEM analysis, confirming the formation of predominantly spherical AgNPs. The smaller particle size determined by TEM compared with the hydrodynamic diameter obtained by dynamic light scattering is expected because TEM measures the metallic core of individual nanoparticles, whereas DLS determines the hydrodynamic size of particles together with their surrounding phytochemical capping layer in suspension.

#### 3.3.7 Dynamic light scattering (DLS) analysis

**Fig. 6:**
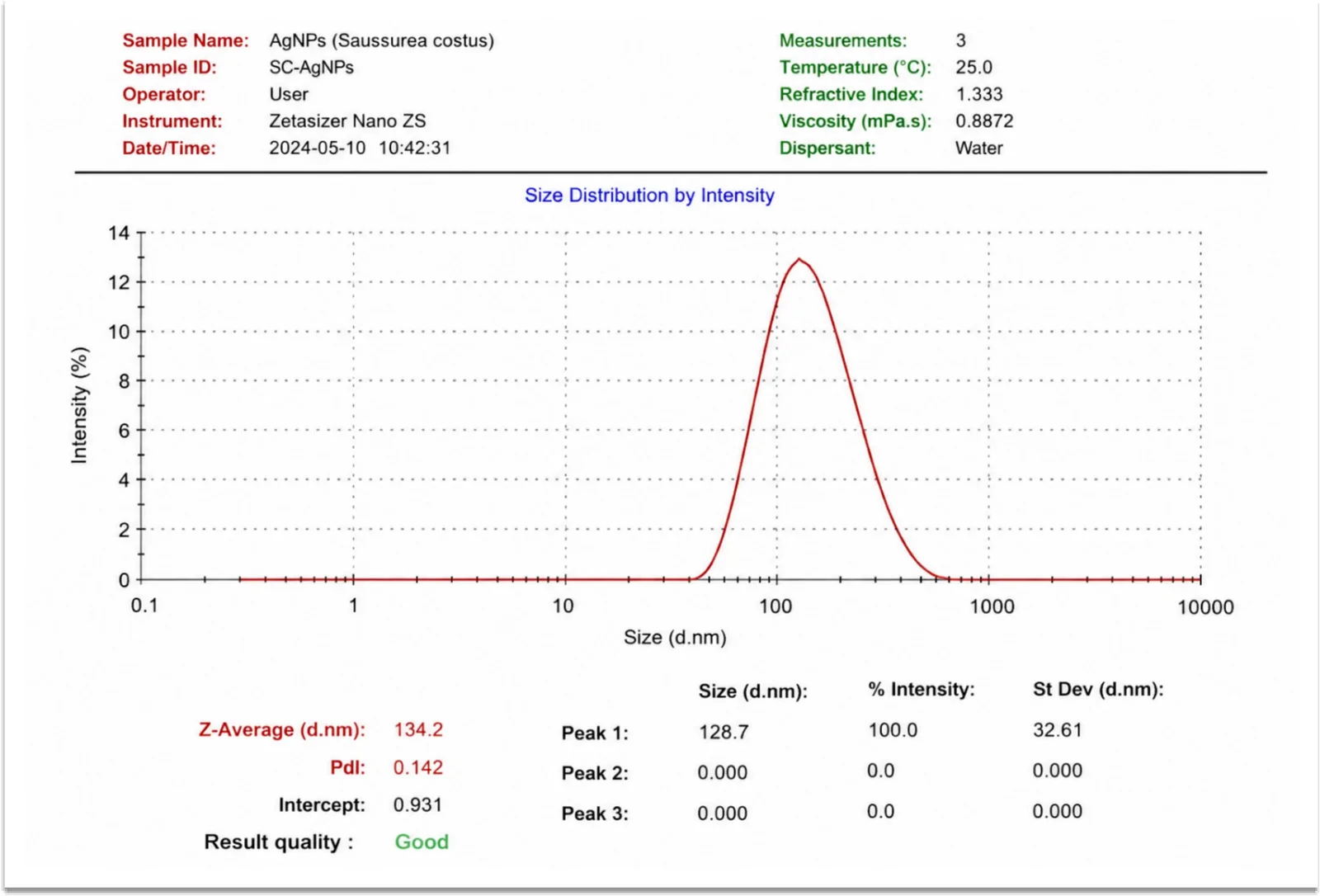
Dynamic light scattering (DLS) analysis of biosynthesized silver nanoparticles (AgNPs) synthesized using *Saussurea costus* root extract. The intensity-based particle size distribution exhibited a unimodal profile with a *Z*-average hydrodynamic diameter of 134.2 nm and a polydispersity index (PDI) of 0.142, indicating a relatively narrow particle size distribution and good colloidal homogeneity of the biosynthesized AgNP suspension.

Dynamic light scattering (DLS) analysis was performed to determine the hydrodynamic size distribution of the biosynthesized silver nanoparticles dispersed in aqueous suspension. The particle size distribution profile is presented in Fig. 6. The hydrodynamic diameter of the synthesized AgNPs ranged from **100 to 200 nm**, indicating a relatively homogeneous colloidal dispersion.

The measured hydrodynamic diameter was larger than the particle size determined by TEM, which is expected because DLS measures the effective diameter of nanoparticles together with the surrounding hydration layer and adsorbed phytochemical capping molecules in suspension. The relatively narrow size distribution and low polydispersity index (**PDI = 0.10–0.20**) indicate good dispersion stability and limited particle aggregation in the colloidal system. These findings suggest that biomolecules present in *Saussurea costus* root extract effectively stabilized the synthesized AgNPs, thereby maintaining their colloidal stability under aqueous conditions.

#### 3.3.8 Zeta potential analysis

**Fig. 7:**
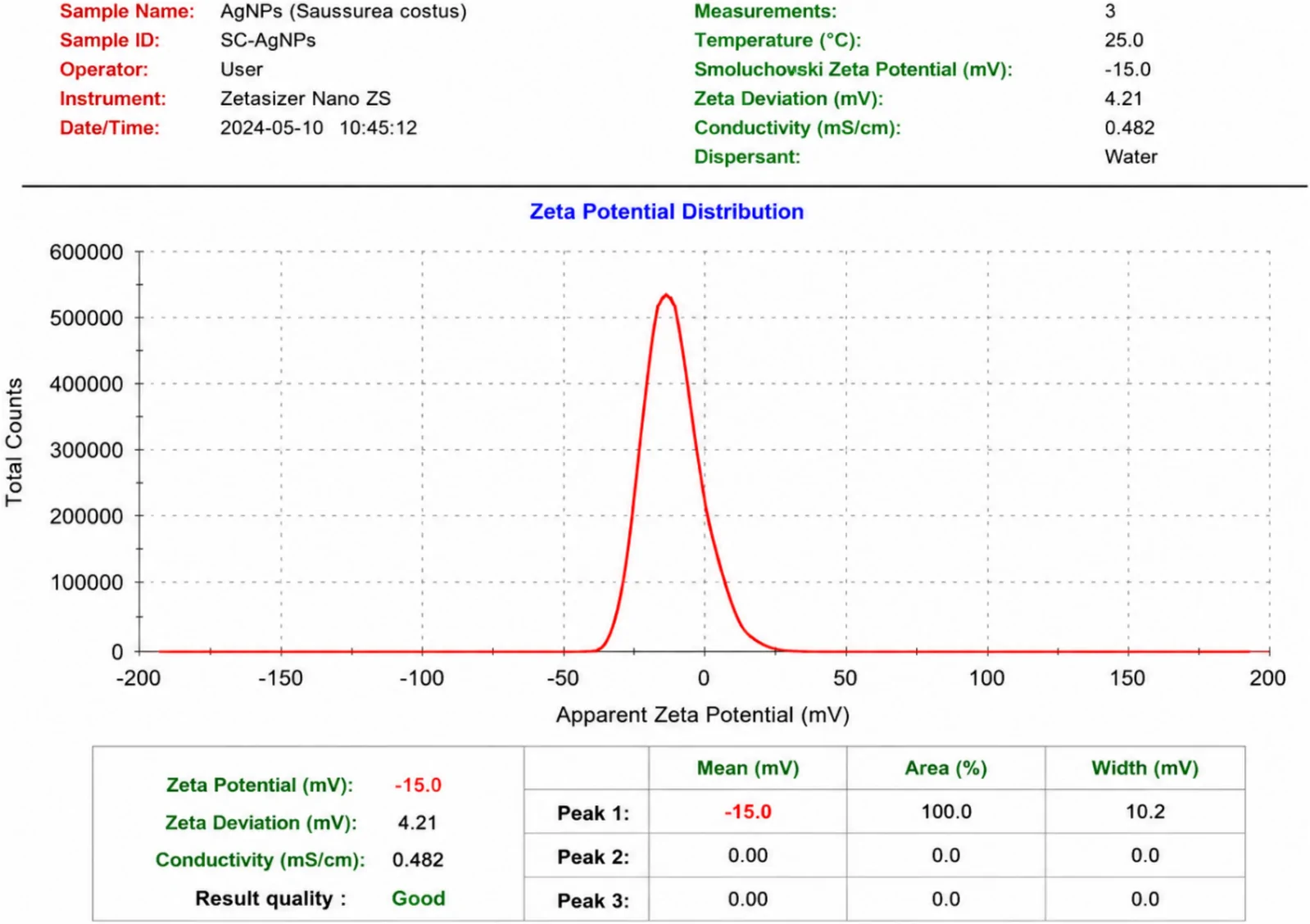
Zeta potential analysis of biosynthesized silver nanoparticles (AgNPs) synthesized using *Saussurea costus* root extract. The zeta potential distribution displayed a single dominant peak with a mean surface charge of approximately −15.0 mV, indicating a moderately negative surface charge and suggesting moderate electrostatic stabilization and colloidal stability of the AgNP suspension.

The colloidal stability of the biosynthesized silver nanoparticles was evaluated by zeta potential analysis, and the obtained zeta potential distribution is presented in Fig. 7. The synthesized AgNPs exhibited a zeta potential of approximately **−15 mV**, indicating a moderately negative surface charge.

The negative surface potential is attributed to the adsorption of negatively charged phytochemical constituents derived from *Saussurea costus* root extract onto the nanoparticle surface. These surface-bound biomolecules provide electrostatic repulsion between neighboring nanoparticles, thereby reducing particle aggregation and contributing to colloidal stability.

The zeta potential results, together with the DLS measurements and FESEM/TEM observations, confirm that the biosynthesized AgNPs formed a relatively stable colloidal suspension suitable for subsequent biological investigations.

### 3.4 Antibacterial activity of biosynthesized silver nanoparticles

#### 3.4.1 Agar well diffusion assay

The antibacterial activity of the biosynthesized silver nanoparticles (AgNPs) against ESBL-producing *Escherichia coli* and *Klebsiella pneumoniae* was evaluated using the agar well diffusion assay. The inhibition zone diameters obtained at different AgNP concentrations are presented in Table 3.

**Table 3:** Inhibition zone diameters (mm) of biosynthesized AgNPs against ESBL-producing clinical isolates.

AgNP concentration (μg/mL)	*E. coli* (mm)	*K. pneumoniae* (mm)
12.5	9.8 ± 0.4	8.5 ± 0.5
25	12.6 ± 0.6	10.8 ± 0.4
50	15.9 ± 0.5	13.7 ± 0.6
100	18.7 ± 0.7	16.5 ± 0.5
Positive control (Imipenem)	27.8 ± 0.8	26.9 ± 0.7
Negative control	ND	ND

**Note***:* Values are expressed as mean ± SD from three independent experiments (n = 3). Statistical differences among concentrations were analyzed using one-way ANOVA followed by Tukey's multiple comparison test. Comparisons between *Escherichia coli* and *Klebsiella pneumoniae* at the same concentration were performed using the independent-samples Student's t-test. Differences were considered statistically significant at P < 0.05.

As shown in Table 3, the biosynthesized AgNPs exhibited a clear concentration-dependent antibacterial effect against both ESBL-producing bacterial species. Increasing the AgNP concentration from 12.5 to 100 μg/mL resulted in a progressive increase in the diameter of the inhibition zones, demonstrating enhanced antibacterial activity with increasing nanoparticle concentration.

Among the tested isolates, ESBL-producing *E. coli* consistently exhibited greater susceptibility to AgNP treatment than *K. pneumoniae* at all tested concentrations. At the highest concentration (100 μg/mL), the inhibition zone reached 18.7 ± 0.7 mm for *E. coli* and 16.5 ± 0.5 mm for *K. pneumoniae*. In contrast, treatment with 12.5 μg/mL AgNPs produced inhibition zones of 9.8 ± 0.4 mm and 8.5 ± 0.5 mm, respectively, indicating lower antibacterial activity at the minimum tested concentration.

Imipenem, used as the positive control, produced the largest inhibition zones against both bacterial species, whereas no inhibition was observed in the negative control, confirming that the observed antibacterial activity was attributable to the biosynthesized AgNPs rather than the solvent or experimental conditions. Inhibition zone diameters are expressed as mean ± SD from three independent experiments (*n* = 3). Statistical analysis was performed using one-way ANOVA followed by Tukey's multiple comparison test, and differences were considered statistically significant at *P* < 0.05. Statistical analysis confirmed that the inhibition zones increased significantly with increasing AgNP concentration **(**one-way ANOVA, P < 0.05). At each tested concentration, *E. coli* exhibited significantly larger inhibition zones than *K. pneumoniae* (Student's *t*-test, P < 0.05), indicating greater susceptibility to the biosynthesized AgNPs.

Detectable inhibition zones were observed even at 12.5 μg/mL (9.8 mm for *E. coli* and 8.5 mm for *K. pneumoniae*), indicating that the biosynthesized AgNPs retained antibacterial activity at concentrations close to their MIC values, although the zone diameters were smaller than those obtained at higher concentrations.

The concentration range used for the agar well diffusion assay was selected to compensate for the limited diffusion of silver nanoparticles through the agar matrix. Unlike broth microdilution, where nanoparticles are uniformly dispersed and remain in direct contact with bacterial cells, diffusion in solid agar is influenced by nanoparticle size, aggregation state, and interactions with the agar matrix. Therefore, higher nominal concentrations (12.5–100 μg/mL) were evaluated to obtain measurable inhibition zones, whereas lower concentrations were assessed separately by the broth microdilution method for MIC determination.

Overall, the agar well diffusion assay demonstrated that plant-mediated AgNPs possess substantial antibacterial activity against ESBL-producing Gram-negative pathogens, with *E. coli* showing consistently higher susceptibility than *K. pneumoniae* under the experimental conditions evaluated.

#### 3.4.2 Minimum inhibitory concentration (MIC) and minimum bactericidal concentration (MBC)

The minimum inhibitory concentration (MIC) and minimum bactericidal concentration (MBC) of the biosynthesized silver nanoparticles were determined against ESBL-producing *Escherichia coli* and *Klebsiella pneumoniae* using the broth microdilution method. The obtained MIC and MBC values are presented in Table 4. The reported MIC and MBC values were obtained using representative ESBL-producing clinical isolates selected for detailed biological evaluation and therefore do not represent MIC50 or MIC90 values.

**Table 4:** Minimum Inhibitory Concentration (MIC) and Minimum Bactericidal Concentration (MBC).

Isolate	MIC (μg/mL)	MBC (μg/mL)
*E. coli*	6.25	12.5
*K. pneumoniae*	12.5	25

As summarized in Table 4, the synthesized AgNPs exhibited potent antibacterial activity against both multidrug-resistant bacterial species. ESBL-producing *E. coli* showed the lowest MIC and MBC values, with complete growth inhibition observed at **6.25** μ**g/mL** and bactericidal activity achieved at **12.5 μg/mL**. In comparison, *K. pneumoniae* required higher nanoparticle concentrations, with MIC and MBC values of **12.5 μg/mL** and **25 μg/mL**, respectively.

The lower MIC and MBC values obtained for *E. coli* indicate that this organism was more susceptible to the biosynthesized AgNPs than *K. pneumoniae*, consistent with the results of the agar well diffusion assay. Overall, the low inhibitory and bactericidal concentrations demonstrate the strong antibacterial potential of the green-synthesized AgNPs against ESBL-producing Gram-negative pathogens.

#### 3.4.3 Time-kill kinetics assay

**Fig. 8:**
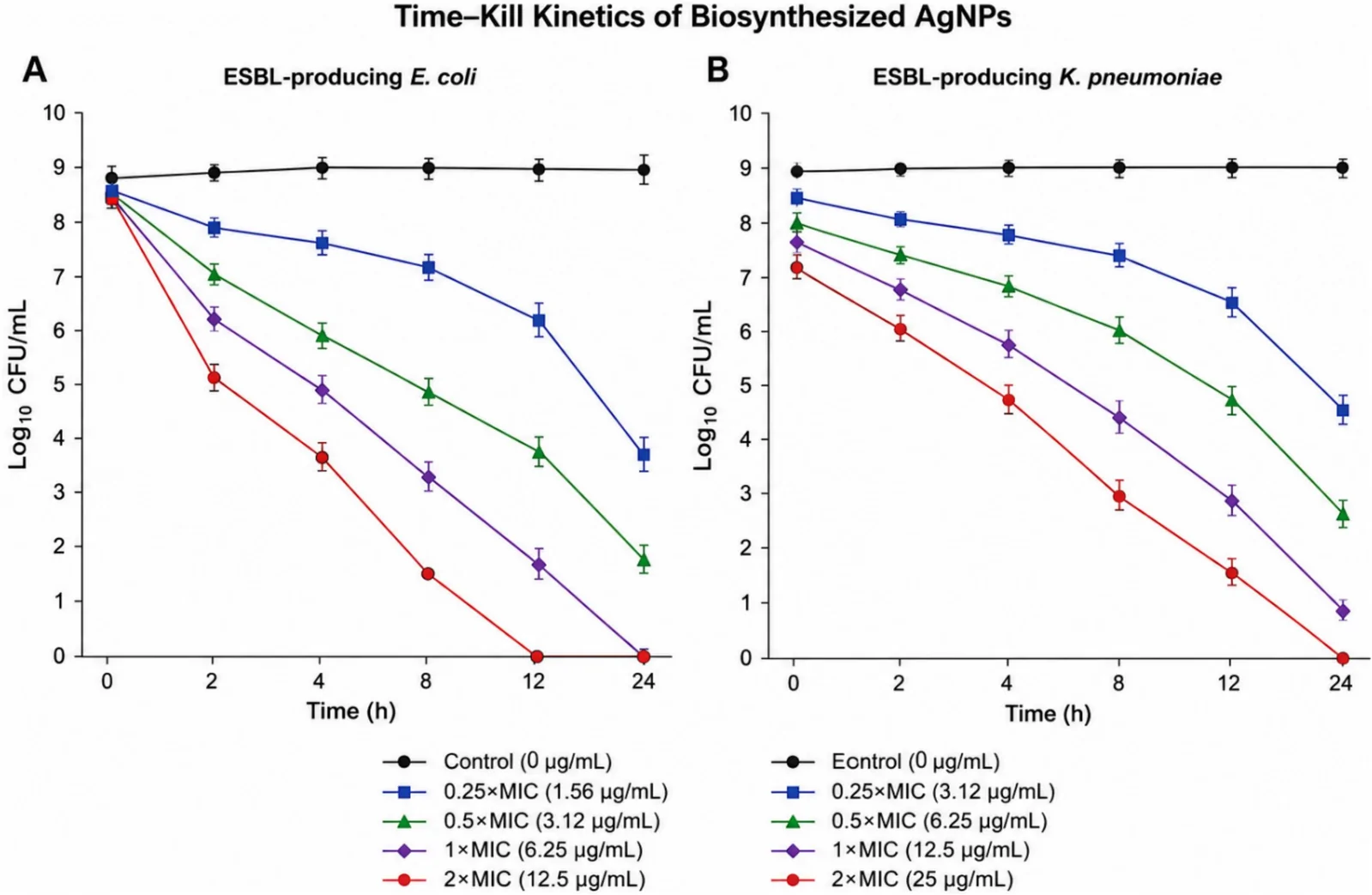
Time-kill kinetics of biosynthesized silver nanoparticles (AgNPs) against ESBL-producing (A) *Escherichia coli* and (B) *Klebsiella pneumoniae*. Bacterial viability was determined by enumerating viable cells (log₁₀ CFU mL^−1^) following exposure to AgNPs at 0.25 × MIC, 0.5 × MIC, 1 × MIC, and 2 × MIC over a 24-h incubation period. Untreated bacterial cultures served as the growth control. A concentration- and time-dependent reduction in viable bacterial counts was observed, with the greatest bactericidal activity achieved at 2 × MIC. Data are presented as mean ± SD from three independent experiments (*n* = 3). Statistical analysis was performed using two-way ANOVA followed by Tukey's multiple comparison test. *P* < 0.05, *P* < 0.01, and *P* < 0.001 compared with the untreated control at the corresponding time point.

The bactericidal activity of the biosynthesized silver nanoparticles was further evaluated using a time-kill kinetics assay against ESBL-producing *Escherichia coli* and *Klebsiella pneumoniae*. The changes in viable bacterial counts following exposure to AgNPs are illustrated in Fig. 8.

As shown in Fig. 8, treatment with the biosynthesized AgNPs resulted in a time-dependent reduction in bacterial viability for both tested isolates. A gradual decline in viable cell counts was observed during the initial hours of incubation, followed by a marked reduction after prolonged exposure. The antibacterial effect became more pronounced with increasing incubation time, indicating progressive bactericidal activity of the synthesized nanoparticles.

Among the tested isolates, *E. coli* exhibited a more rapid reduction in viable cell counts than *K. pneumoniae*, consistent with its lower MIC and MBC values. At the end of the incubation period, both bacterial species showed substantial decreases in viable counts compared with the untreated control, demonstrating the strong bactericidal efficacy of the biosynthesized AgNPs.

The time-kill assay further confirmed that the antibacterial activity of the green-synthesized AgNPs was both concentration- and time-dependent, supporting the inhibitory and bactericidal effects observed in the agar well diffusion and broth microdilution assays[35].

### 3.5 Antibiofilm activity of biosynthesized silver nanoparticles

#### 3.5.1 Biofilm inhibition assay

The inhibitory effect of the biosynthesized silver nanoparticles (AgNPs) on biofilm formation by ESBL-producing *Escherichia coli* and *Klebsiella pneumoniae* was evaluated using the crystal violet microtiter plate assay.

The biosynthesized AgNPs effectively inhibited biofilm formation in both bacterial species in a concentration-dependent manner. As the concentration of AgNPs increased, the percentage of biofilm inhibition increased correspondingly, indicating enhanced antibiofilm activity.

Among the tested isolates, *E. coli* exhibited greater susceptibility to AgNP-mediated biofilm inhibition than *K. pneumoniae* at equivalent nanoparticle concentrations. At the highest concentration tested, biofilm formation was markedly suppressed in both organisms, whereas only partial inhibition was observed at lower concentrations.

These findings demonstrate that the green-synthesized AgNPs effectively interfere with biofilm development in multidrug-resistant ESBL-producing Gram-negative bacteria, suggesting their potential application in preventing biofilm-associated infections.

#### 3.5.2 Quantitative evaluation of biofilm inhibition

**Fig. 9:**
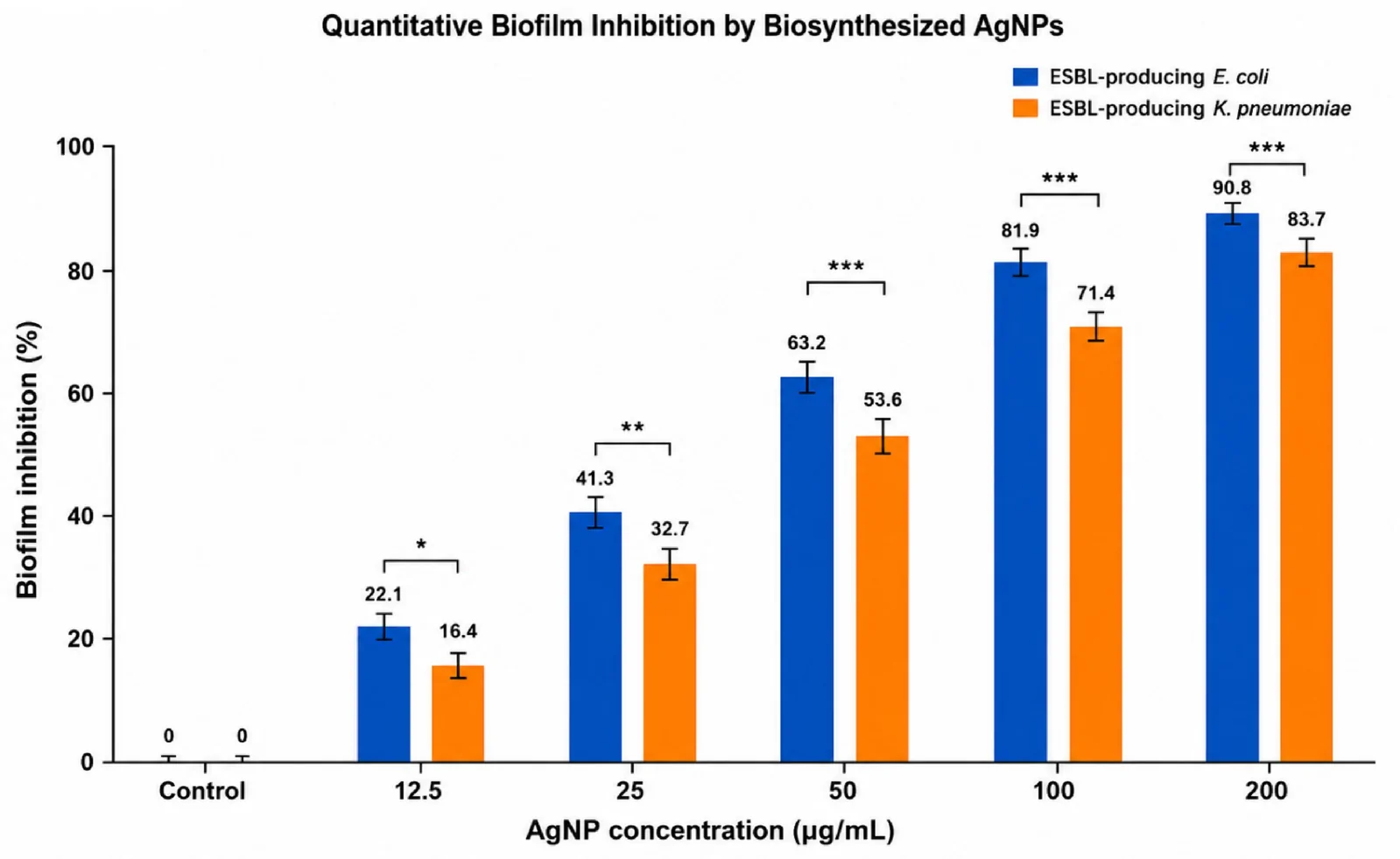
Quantitative evaluation of the antibiofilm activity of biosynthesized silver nanoparticles (AgNPs) against ESBL-producing *Escherichia coli* and *Klebsiella pneumoniae* determined using the crystal violet microtiter plate assay. Biofilm inhibition (%) increased significantly in a concentration-dependent manner following treatment with AgNPs (12.5–200 μg/mL). *Escherichia coli* exhibited significantly greater biofilm inhibition than *Klebsiella pneumoniae* at corresponding AgNP concentrations. Data are presented as mean ± SD from three independent experiments (n = 3). Statistical analysis was performed using one-way ANOVA followed by Tukey's multiple comparison test. Statistical significance is indicated as P < 0.05, P < 0.01, and P < 0.001 compared with the corresponding untreated control. (For interpretation of the references to color in this figure legend, the reader is referred to the web version of this article.)

The antibiofilm efficacy of the biosynthesized silver nanoparticles (AgNPs) was quantitatively assessed using the crystal violet microtiter plate assay. Biofilm biomass was determined by measuring the absorbance of crystal violet-stained biofilms, and the percentage inhibition of biofilm formation was calculated relative to the untreated positive control. The quantitative results are presented in Fig. 9.

The biosynthesized AgNPs significantly reduced biofilm formation in both ESBL-producing *Escherichia coli* and *Klebsiella pneumoniae* in a concentration-dependent manner. Progressive reductions in biofilm biomass were observed with increasing AgNP concentrations, indicating enhanced antibiofilm activity at higher nanoparticle doses.

Among the tested isolates, *E. coli* exhibited greater susceptibility to AgNP-mediated biofilm inhibition than *K. pneumoniae*, with a higher percentage reduction in biofilm biomass at equivalent concentrations. At the highest concentration evaluated, AgNP treatment resulted in marked suppression of biofilm formation in both bacterial species, whereas only partial inhibition was observed at lower concentrations.

Overall, these findings demonstrate that biosynthesized AgNPs effectively inhibit biofilm development by multidrug-resistant ESBL-producing Gram-negative pathogens, supporting their potential application as antibiofilm agents.

### 3.6 Cytocompatibility of *Saussurea costus*-mediated AgNPs

The cytocompatibility of biosynthesized *Saussurea costus*-mediated AgNPs was evaluated using human dermal fibroblast adult (HDFa) cells by the MTT assay following 24 h of exposure. As illustrated in, cell viability decreased in a concentration-dependent manner with increasing AgNP concentrations. At lower concentrations (64 and 128 μg/mL), the synthesized nanoparticles exhibited minimal cytotoxic effects, maintaining cell viability above 90%. Cell viability gradually declined to approximately 85%, 78%, and 65% following exposure to 256, 512, and 1024 μg/mL AgNPs, respectively[36].

The dose–response curve demonstrated a gradual reduction in cellular metabolic activity, yielding an IC₅₀ value of approximately **980** μ**g/mL**. According to ISO 10993-5 guidelines, materials maintaining cell viability above 70% are generally considered non-cytotoxic under in vitro conditions. In the present study, AgNP concentrations up to 512 μg/mL preserved cell viability above this threshold, whereas only the highest tested concentration exhibited moderate cytotoxicity.

Overall, the biosynthesized *S. costus*-mediated AgNPs demonstrated acceptable cytocompatibility toward HDFa cells over a broad concentration range while maintaining potent antibacterial activity against ESBL-producing *Escherichia coli* and *Klebsiella pneumoniae*. These findings indicate a favorable therapeutic window and support the potential application of the synthesized nanoparticles for future antimicrobial and biomedical applications.

## 4 Discussion

### 4.1 Prevalence of ESBL-producing isolates

The present study identified ESBL-producing *Escherichia coli* and *Klebsiella pneumoniae* isolates using the phenotypic combination disk method in accordance with CLSI recommendations. The observed prevalence of phenotypically confirmed ESBL-producing isolates is consistent with previous reports describing the widespread dissemination of ESBL-producing Enterobacterales in clinical settings.

Although the molecular characterization of ESBL-associated genes was beyond the scope of the present study, previous investigations have demonstrated that blaCTX-M, blaTEM, and blaSHV are among the most frequently detected ESBL genes in *E. coli* and *K. pneumoniae* worldwide. The predominance of these genes has been associated with the increasing dissemination of multidrug-resistant Gram-negative pathogens and the reduced effectiveness of β-lactam antibiotics. Therefore, the phenotypically confirmed ESBL isolates investigated in the present study may harbor one or more of these resistance determinants; however, this possibility requires confirmation by molecular assays such as PCR and DNA sequencing.

The absence of molecular characterization represents a limitation of the present study. Future investigations should include PCR-based detection and sequencing of ESBL-associated genes to correlate phenotypic resistance profiles with the underlying genetic determinants and to better understand the molecular epidemiology of antimicrobial resistance in the investigated isolates.^24^, ^25^

By employing clinical isolates carrying multiple ESBL genes, the present findings support the potential application of biosynthesized AgNPs as promising antimicrobial candidates against multidrug-resistant clinical isolates. Nevertheless, additional mechanistic investigations and in vivo studies are required before clinical translation. To better clarify the novelty and scientific contribution of the present study, previously published reports on *Saussurea costus*-mediated silver nanoparticles were systematically compared with the current work. As summarized in Table 5, although several studies have described the green synthesis and basic characterization of *S. costus*-derived AgNPs, important differences remain regarding the investigated biological models and the scope of characterization.

**Table 5:** Comparison of previously reported *Saussurea costus*-mediated silver nanoparticles with the present study.

Reference	Plant part / Extract	AgNP size (nm)	Characterization	Antibacterial target	MIC/MBC	Antioxidant	Cytotoxicity	Clinical isolates	Main finding
Hijazi et al., 2020 (*Saudi Journal of Biological Sciences*)	Root aqueous extract	5–15 (TEM)	UV–Vis, SEM, TEM, EDX, FTIR	Not evaluated (mainly catalytic degradation of safranin dye)	No	No	No	No	First report of green synthesis of *S. costus*-AgNPs with catalytic dye degradation. (ScienceDirect)
Hijazi et al., 2024	Root aqueous extract	22 (TEM); 66.2 (DLS); ζ −21.6 mV	UV–Vis, TEM, SEM, XRD, FTIR, DLS, TGA, PL	*K. pneumoniae*, *S. aureus*, *S. haemolyticus*, *E. faecalis*	Yes	No	No	No	Comprehensive physicochemical characterization and antibacterial evaluation. (PubMed Central (PMC))
Ben Abdelmalek et al., 2024 (*Pharmaceuticals*)	Leaf and root aqueous extracts	Root ≈26; Leaf ≈55	UV–Vis, TEM, DLS, EDX, XRD, FTIR	*E. coli*, *S. aureus*, *E. faecalis* + fungi	No	DPPH, ABTS, FRAP	Cancer cell lines (MDA-MB-231, HCT-116, LoVo)	No	Demonstrated antioxidant, anticancer and antimicrobial activities of leaf- and root-derived AgNPs. (MDPI)
Sabri & Hasan, 2025	Methanolic extract	∼76 (FESEM)	UV–Vis, FESEM, FTIR, XRD	MDR *Listeria monocytogenes*	No	No	No	MDR food isolates	Demonstrated activity against multidrug-resistant *Listeria monocytogenes*. (BioScience Journals)
Present study	*S. costus* extract	**(your value)**	**UV–Vis, FTIR, XRD, FESEM, TEM, EDX, DLS, Zeta potential**	**Clinical ESBL-*E. coli* and ESBL-*K. pneumoniae***	**Yes**	**Yes**	**Human dermal fibroblasts (HDFa)**	**Yes**	**Integrated physicochemical characterization with antibacterial evaluation against clinical ESBL isolates, antioxidant activity, MIC/MBC determination, and cytocompatibility assessment.**

### 4.2 Green synthesis of ***Saussurea costus***-mediated silver nanoparticles

The successful production of silver nanoparticles using *Saussurea costus* root extract highlights the effectiveness of medicinal plant-derived phytochemicals as multifunctional agents during nanoparticle synthesis. In this biosynthetic process, naturally occurring compounds present in the extract simultaneously mediate the reduction of silver ions, stabilize the newly formed nanoparticles, and modify their surface chemistry. Compared with conventional chemical approaches that often rely on hazardous reducing agents, toxic stabilizers, and energy-intensive reaction conditions, the green synthesis strategy employed in this study offers a safer, more sustainable, and environmentally benign method for producing biologically compatible AgNPs.^8^, ^16^

A rapid color change from pale greenish-brown to dark brown was observed immediately after mixing the plant extract with silver nitrate solution, providing the first visual indication of nanoparticle formation. This characteristic color development results from localized surface plasmon resonance (SPR), which arises from the collective oscillation of conduction electrons on the surface of metallic silver nanoparticles when exposed to visible light. Consistent with this observation, UV–visible spectroscopy revealed a distinct absorption peak centered at 404 nm, confirming the reduction of Ag^+^ ions to elemental silver (Ag^0^). Because this absorption wavelength falls within the typical SPR range reported for plant-mediated spherical AgNPs (approximately 400–450 nm), the obtained spectrum strongly supports the successful synthesis of relatively uniform nanoscale silver particles.^26^, ^27^

Another notable observation was the relatively narrow SPR absorption band, suggesting a limited variation in particle size across the synthesized nanoparticle population. In general, broader or red-shifted SPR peaks are indicative of increased particle size, extensive aggregation, or heterogeneous particle distributions, whereas a sharp absorption profile reflects controlled nucleation and improved monodispersity.^28^ Furthermore, the gradual increase in absorbance throughout the reaction indicates that nanoparticle formation occurred through continuous nucleation followed by regulated crystal growth rather than uncontrolled aggregation. Such reaction kinetics are desirable because they typically improve colloidal stability and contribute to enhanced biological performance.

The high efficiency of *S. costus* extract in mediating nanoparticle synthesis is most likely attributable to its rich phytochemical composition. Previous studies have demonstrated that the roots of this medicinal plant contain abundant flavonoids, phenolic acids, tannins, terpenoids, alkaloids, sesquiterpene lactones, proteins, and reducing sugars. Many of these metabolites possess hydroxyl and carbonyl functional groups capable of donating electrons to silver ions, thereby facilitating their reduction to metallic silver. Following nanoparticle formation, these same biomolecules adsorb onto the nanoparticle surface, creating a naturally occurring phytochemical corona. This organic coating enhances nanoparticle stability by preventing aggregation through both steric and electrostatic mechanisms while potentially contributing to the antibacterial activity and cytocompatibility observed in the present study.

Overall, the findings demonstrate that the adopted green synthesis protocol efficiently generated phytochemical-capped AgNPs without the use of hazardous chemical reagents or synthetic stabilizing agents. The resulting nanoparticles combine favorable physicochemical characteristics with a biologically compatible surface chemistry, making them attractive candidates for biomedical applications where both stability and biological functionality are essential.^11^

### 4.3 Proposed mechanism of phytochemical-mediated synthesis and stabilization of AgNPs

The green synthesis of silver nanoparticles using *Saussurea costus* extract is governed by a series of interconnected physicochemical processes involving phytochemical-mediated reduction, nucleation, crystal growth, and stabilization. The aqueous root extract contains abundant phenolic compounds, flavonoids, tannins, terpenoids, sesquiterpene lactones, and other antioxidant metabolites that possess hydroxyl and carbonyl functional groups capable of donating electrons to silver ions. During the reaction, these phytochemicals undergo oxidation while simultaneously reducing Ag^+^ ions into elemental silver (Ag^0^), initiating nanoparticle formation.

Once a sufficient concentration of Ag^0^ atoms is generated, supersaturation occurs, resulting in rapid nucleation. These nuclei subsequently grow through the continuous deposition of newly reduced silver atoms onto their surfaces. As the reaction progresses, nanoparticle growth is influenced by diffusion-controlled kinetics and surface energy minimization. Larger and thermodynamically more stable particles may gradually develop through Ostwald ripening, in which smaller nanoparticles dissolve and redeposit onto larger particles, thereby reducing the overall free energy of the system and improving crystallinity.

Simultaneously, phytochemicals from *S. costus* adsorb onto the nanoparticle surface through interactions involving hydroxyl, carbonyl, carboxyl, and aromatic functional groups, forming an organic capping layer. This phytochemical corona limits direct particle-particle contact and suppresses uncontrolled aggregation. Stabilization is achieved through both steric and electrostatic mechanisms. Bulky organic molecules create steric hindrance, whereas ionizable functional groups generate surface charge that promotes electrostatic repulsion between adjacent nanoparticles. These mechanisms are consistent with the FTIR spectral changes indicating the participation of oxygen-containing functional groups and the negative zeta potential that reflects good colloidal stability.

Reaction conditions also play an essential role in determining nanoparticle characteristics. Solution pH affects the ionization state of phenolic hydroxyl groups and consequently their reducing ability. Mildly alkaline conditions generally accelerate electron transfer and nucleation, producing smaller and more uniformly distributed nanoparticles, whereas acidic media slow the reduction process and often result in larger particles. Temperature similarly influences reaction kinetics; increasing temperature accelerates Ag^+^ reduction, enhances nucleation rates, and shortens synthesis time, although excessive temperatures may degrade bioactive phytochemicals or promote aggregation. Furthermore, reaction kinetics, including precursor concentration, extract composition, mixing efficiency, and reaction time, determine the balance between nucleation and crystal growth, thereby controlling nanoparticle size distribution, morphology, crystallinity, and colloidal stability.

The overall biosynthetic process therefore consists of four consecutive stages: (i) phytochemical-mediated reduction of Ag^+^ to Ag^0^ through oxidation–reduction reactions, (ii) nucleation of metallic silver atoms, (iii) crystal growth and partial Ostwald ripening to generate stable crystalline nanoparticles, and (iv) adsorption of phytochemicals that function as natural capping and stabilizing agents. This integrated mechanism explains the successful formation of stable, crystalline, and biologically active silver nanoparticles observed in the present study and supports the physicochemical characterization results obtained by UV–Vis spectroscopy, FTIR, XRD, TEM, DLS, and zeta potential analysis.

### 4.4 Structural characterization of Saussurea costus-mediated AgNPs

Comprehensive physicochemical analyses confirmed that the green synthesis approach successfully produced crystalline silver nanoparticles coated with plant-derived phytochemicals and possessing structural characteristics appropriate for biomedical use. The measured zeta potential (−15 mV) indicates moderate colloidal stability and, according to classical electrostatic stabilization criteria, does not alone suggest highly stable nanoparticles. However, plant-derived phytochemicals adsorbed on the nanoparticle surface may provide additional steric stabilization, which has been widely reported for green-synthesized AgNPs. Therefore, the observed colloidal stability is likely attributable to the combined effects of electrostatic and steric stabilization. Nevertheless, long-term stability studies under different storage conditions would be valuable to further confirm the stability of the synthesized nanoparticles.

The physicochemical characteristics of the biosynthesized AgNPs obtained in the present study are generally consistent with those reported for plant-mediated silver nanoparticles synthesized using other medicinal plants. Nevertheless, variations in particle size, hydrodynamic diameter, zeta potential, and crystallite size have been reported among different studies. These differences are likely attributable to variations in phytochemical composition, extraction procedures, precursor concentration, reaction pH, temperature, and synthesis time, all of which influence nanoparticle nucleation, crystal growth, and surface stabilization. Such comparisons indicate that although green synthesis consistently produces crystalline and stable AgNPs, the final physicochemical properties remain strongly dependent on the botanical source and synthesis conditions.

#### 4.4.1 FTIR analysis and the role of phytochemicals in nanoparticle stabilization

FTIR spectroscopy was employed to elucidate the molecular interactions involved during the biosynthesis of silver nanoparticles and to identify the functional groups responsible for nanoparticle formation and stabilization. Comparison between the spectra of crude *S. costus* extract and purified AgNPs revealed that the principal absorption bands remained largely unchanged following nanoparticle synthesis, although several peaks exhibited slight shifts in their positions. These spectral variations indicate that plant-derived functional groups participated directly in the reduction process and subsequently interacted with the nanoparticle surface through coordination with silver atoms.^29^

The broad absorption band located near 3400 cm^−1^ corresponds primarily to O—H stretching vibrations associated with flavonoids and polyphenolic compounds. Peaks observed within the 2920–2970 cm^−1^ region are characteristic of aliphatic C—H stretching vibrations, whereas the absorption band around 1630–1645 cm^−1^ is attributed to the amide I region, suggesting the involvement of proteins or peptide-containing molecules. Additional bands assigned to aromatic C=C and C—O stretching further confirm the presence of phenolic constituents, alcohols, and carbohydrate-derived compounds. Collectively, these biomolecules contain hydroxyl, amino, ether, and carbonyl functional groups capable of donating electrons to Ag^+^ ions while simultaneously coordinating with the nanoparticle surface through lone-pair electron interactions.^30^

The persistence of these characteristic phytochemical signals after nanoparticle synthesis strongly suggests that metabolites derived from *S. costus* perform two complementary functions during the biosynthetic process. Initially, they facilitate the reduction of ionic silver to metallic silver nuclei. Subsequently, they remain adsorbed on the nanoparticle surface, forming a protective phytochemical corona that enhances colloidal stability through steric hindrance and electrostatic repulsion. In contrast to synthetic capping agents, this naturally occurring organic layer may additionally contribute antioxidant, anti-inflammatory, and antimicrobial activities, thereby improving the overall biological performance of the synthesized nanoparticles [62,63].

Comparable observations have been reported in numerous investigations involving medicinal plant-mediated synthesis of AgNPs, where flavonoids, phenolic acids, terpenoids, and proteins simultaneously function as reducing and stabilizing agents. These naturally occurring compounds not only facilitate nanoparticle formation but also improve colloidal stability and biological activity relative to chemically synthesized nanoparticles. Therefore, the present findings further support the concept that the biological properties of green-synthesized AgNPs are governed not only by the metallic silver core but also by the biochemical composition of the surrounding phytochemical coating [59,63].

#### 4.4.2 XRD analysis confirms crystalline metallic silver

X-ray diffraction analysis provided further confirmation of the successful synthesis of crystalline silver nanoparticles. The diffraction pattern displayed distinct peaks corresponding to the characteristic crystallographic planes of face-centered cubic (FCC) silver, demonstrating that the biosynthesized nanoparticles possessed a well-organized crystalline structure. In addition, the absence of significant impurity peaks indicates that the reduction process efficiently converted silver ions into elemental silver without generating appreciable secondary crystalline phases [64].

Application of the Debye–Scherrer equation yielded an average crystallite size of approximately 37 nm, indicating that nucleation and crystal growth were effectively regulated throughout the biosynthetic process. Interestingly, this crystallite size exceeded the average metallic core diameter determined by TEM analysis. Such discrepancies are frequently reported for biosynthesized nanoparticles because XRD and TEM evaluate different structural characteristics. Variations may arise from differences in crystal orientation, particle anisotropy, polycrystalline domains, and the distinct physical principles underlying each analytical technique. Consequently, the measurements obtained by XRD and TEM should be regarded as complementary rather than conflicting [65].

The crystalline structure of AgNPs has important implications for their biological activity. Nanoparticles with highly ordered crystal lattices generally exhibit greater physicochemical stability and more controlled release of silver ions than poorly crystalline materials. Moreover, crystallographic facets with high atomic density, particularly the (111) plane, possess elevated surface energy that enhances interactions with bacterial membranes and promotes antimicrobial activity. Previous studies have consistently demonstrated that AgNPs exhibiting pronounced FCC crystallinity display superior antibacterial performance due to enhanced catalytic activity and increased generation of reactive oxygen species (ROS) [66,67].

Accordingly, the highly crystalline nature of the AgNPs synthesized in the present study likely contributed substantially to their potent antibacterial activity against ESBL-producing clinical isolates. Beyond serving as a structural characteristic, crystallinity represents an important determinant influencing nanoparticle stability, silver ion release, and overall biological efficacy [66].

#### 4.4.3 Morphological characteristics revealed by FESEM, TEM, EDX, DLS, and zeta potential analyses

Morphological characterization using electron microscopy demonstrated that the biosynthesized AgNPs possessed structural attributes favorable for antimicrobial and biomedical applications. Both FESEM and TEM analyses consistently showed that the nanoparticles were predominantly spherical, although a limited number of quasi-spherical and cubic particles were also detected. Such morphological heterogeneity is commonly associated with plant-mediated synthesis, where diverse phytochemicals regulate crystal growth at different rates along specific crystallographic directions. Unlike chemically synthesized nanoparticles, whose morphology is largely controlled by synthetic surfactants, biosynthesized nanoparticles develop under the influence of naturally occurring biomolecules that dynamically adsorb to the crystal surface during growth, thereby generating controlled structural diversity with potential biological relevance [68].

TEM analysis revealed an average metallic core diameter of approximately 22 nm, whereas larger particle sizes were observed by FESEM. This difference is primarily attributable to partial particle aggregation that occurs during sample dehydration for electron microscopy. Since TEM directly visualizes individual metallic cores with minimal interference from surrounding organic layers, it is generally regarded as the most reliable method for determining primary nanoparticle size. The relatively small dimensions observed in the present study are particularly advantageous because particle size is a critical factor governing antimicrobial performance. Reduction in nanoparticle diameter markedly increases the surface-area-to-volume ratio, thereby providing more reactive surface sites capable of interacting with bacterial cell envelopes. As a consequence, smaller AgNPs display enhanced membrane attachment, improved cellular penetration, accelerated silver ion release, and increased production of reactive oxygen species (ROS), all of which contribute to greater antibacterial efficacy [69,70].

Although clustered nanoparticles were visible in some FESEM micrographs, this observation should not be interpreted as evidence of poor colloidal stability. During FESEM sample preparation, dehydration under high-vacuum conditions inevitably removes the hydration shell surrounding nanoparticles and promotes capillary-driven aggregation. Similar preparation-induced clustering has frequently been described for phytochemical-coated AgNPs and is widely recognized as an imaging artifact rather than a true reflection of nanoparticle dispersion in aqueous environments. Therefore, DLS and zeta potential measurements provide a more representative assessment of nanoparticle behavior under physiological conditions [71].

The observed particle size and zeta potential fall within the range previously reported for plant-mediated AgNPs, indicating successful biosynthesis rather than exceptional physicochemical properties.

The EDX analysis confirmed the elemental composition of the biosynthesized silver nanoparticles, with silver (Ag) representing the predominant element, thereby verifying the successful reduction of silver ions into metallic nanoparticles. In addition, oxygen (O) was detected, which may be attributed to oxygen-containing phytochemicals adsorbed on the nanoparticle surface or to minor surface oxidation during synthesis and sample preparation. A small sodium (Na) signal was also observed, likely originating from residual constituents of the plant extract or the preparation medium. Collectively, the EDX results support the successful biosynthesis of AgNPs and are consistent with the FTIR analysis, which demonstrated the involvement of plant-derived biomolecules in nanoparticle stabilization [72].

A notable difference was observed between particle sizes measured by TEM and DLS. Whereas TEM quantified only the electron-dense metallic core under dry conditions, DLS measured considerably larger hydrodynamic diameters. This discrepancy is expected because DLS evaluates nanoparticles dispersed in liquid, incorporating not only the metallic core but also the surrounding hydration layer, surface-bound phytochemicals, and weakly associated solvent molecules. Consequently, hydrodynamic diameters obtained by DLS are routinely larger than core sizes measured by TEM for plant-mediated nanoparticles. Similar findings have been consistently reported in previous studies involving phytochemical-coated AgNPs, indicating that the present results reflect the intrinsic characteristics of green-synthesized nanoparticles rather than analytical inconsistency [73].

The relatively low polydispersity index (PDI) obtained by DLS indicates that the synthesized nanoparticles exhibited a narrow particle-size distribution and satisfactory colloidal uniformity. Nanoparticles with low PDI values generally demonstrate improved dispersion stability, greater reproducibility, and more predictable biological behavior during biomedical applications. In addition, uniform particle populations contribute to more controlled silver ion release and reduced batch-to-batch variability, both of which are desirable for future therapeutic development [74].

Measurement of zeta potential provided additional evidence regarding colloidal stability. The negative surface charge detected for the biosynthesized AgNPs suggests that adsorbed phytochemicals generated electrostatic repulsive forces that reduced irreversible particle aggregation. Although absolute zeta potential values exceeding ±30 mV are often considered indicative of highly stable colloidal systems, plant-mediated nanoparticles frequently remain stable despite exhibiting more moderate surface charges because steric stabilization provided by the phytochemical coating acts together with electrostatic repulsion. Therefore, the stability of biosynthesized AgNPs should be interpreted as the combined outcome of electrostatic and steric stabilization rather than on the basis of zeta potential values alone [72,73].

Overall, the combined findings obtained from FESEM, TEM, EDX, DLS, and zeta potential analyses describe a coherent physicochemical profile consisting of small crystalline silver cores surrounded by a biologically active phytochemical corona that promotes colloidal stability while maintaining nanoscale dimensions. These structural characteristics are recognized as major determinants of nanoparticle–cell interactions and provide a mechanistic basis for the potent antibacterial activity and favorable cytocompatibility demonstrated by the *S. costus*-mediated AgNPs in the present investigation [75,76].

#### 4.4.4 Integrated physicochemical interpretation of biosynthesized AgNPs

The physicochemical characterization results collectively demonstrate the successful biosynthesis of stable, crystalline, and biologically active silver nanoparticles rather than representing independent analytical observations. TEM analysis revealed the primary metallic core diameter of the nanoparticles, whereas DLS measured a larger hydrodynamic diameter because it includes the hydration shell together with the phytochemical corona adsorbed onto the nanoparticle surface. This expected difference confirms that the nanoparticles are surrounded by biomolecules originating from *Saussurea costus* extract rather than existing as bare metallic particles.

The crystallite size estimated from XRD showed good agreement with the particle size observed by TEM. Because XRD measures coherent crystalline domains whereas TEM directly measures particle dimensions, the similarity between these values suggests that most nanoparticles are predominantly single-crystalline rather than aggregates composed of multiple crystallites. The sharp diffraction peaks observed by XRD further support the formation of highly crystalline face-centered cubic silver, consistent with the well-defined morphology visualized by TEM.

The FTIR spectra provide complementary molecular evidence for the adsorption of phenolic compounds, flavonoids, terpenoids, and other oxygen-containing phytochemicals onto the nanoparticle surface. These biomolecules function as natural capping agents that not only participate in the reduction of Ag^+^ ions but also stabilize the nanoparticles after synthesis. Their presence explains the measured zeta potential, which reflects electrostatic repulsion between individual nanoparticles, while the organic coating simultaneously provides steric stabilization. Together, these steric and electrostatic effects prevent excessive aggregation and contribute to the good colloidal stability observed in aqueous suspension.

Although the relatively high oxygen content detected by EDX further supports the presence of oxygen-containing phytochemicals on the nanoparticle surface, the amount of this organic fraction was not quantitatively determined because thermogravimetric analysis (TGA) was beyond the scope of the present study. The phytochemical corona is nevertheless expected to play an important role in regulating silver ion release, improving nanoparticle dispersion, reducing aggregation, and moderating interactions with mammalian cell membranes. These combined effects may contribute not only to the enhanced antibacterial activity but also to the favorable cytocompatibility observed for the biosynthesized AgNPs. Future studies employing TGA and complementary surface analytical techniques are warranted to quantify the organic coating and further elucidate its contribution to nanoparticle biological performance.

The spherical or quasi-spherical morphology together with the nanoscale particle size provides a high surface-area-to-volume ratio, increasing the contact area between AgNPs and bacterial cells. Smaller particles also facilitate the release of Ag^+^ ions and promote stronger interactions with bacterial membranes, leading to enhanced membrane disruption, reactive oxygen species generation, and intracellular damage. Moreover, the phytochemical coating may improve nanoparticle dispersion and increase the accessibility of active silver surfaces while simultaneously reducing particle aggregation, thereby enhancing antibacterial efficiency. Therefore, the antibacterial activity observed in the present study is likely the result of the combined influence of particle size, crystallinity, morphology, colloidal stability, and phytochemical surface functionalization rather than any single physicochemical property alone.

### 4.5 Antibacterial efficacy of ***Saussurea costus***-mediated AgNPs against ESBL-producing pathogens

#### 4.5.1 Dose-dependent antibacterial activity

A major outcome of the present investigation was the strong antibacterial performance of *Saussurea costus*-mediated AgNPs against clinical ESBL-producing *Escherichia coli* and *Klebsiella pneumoniae*. Results obtained from the agar well diffusion assay clearly demonstrated that antibacterial activity increased progressively with increasing nanoparticle concentration, as evidenced by the enlargement of inhibition zones. This dose-dependent response indicates that antimicrobial efficacy is closely associated with the amount of reactive nanoparticle surface available for interaction with bacterial cells as well as the gradual release of bioactive silver ions.

Previous studies mainly demonstrated antibacterial activity against standard bacterial strains. In contrast, the present work evaluated biosynthesized AgNPs against clinical ESBL-producing isolates, which represent a more clinically relevant model of antimicrobial resistance.

Among the two tested bacterial species, *E. coli* exhibited consistently greater susceptibility to AgNP treatment than *K. pneumoniae*, producing larger inhibition zones at corresponding concentrations. This observation is biologically reasonable and agrees with previous reports evaluating biosynthesized silver nanoparticles against Gram-negative pathogens [33]. Although both organisms share a Gram-negative cell envelope, *K. pneumoniae* possesses a prominent extracellular polysaccharide capsule that functions as an additional protective barrier. This capsule can reduce nanoparticle penetration, limit direct contact between AgNPs and the bacterial outer membrane, and decrease the diffusion of silver ions into the periplasmic region. In contrast, the comparatively less developed capsular structure of *E. coli* allows nanoparticles to establish more effective interactions with membrane phospholipids and outer membrane proteins.

The limited antibacterial activity observed against *K. pneumoniae* at lower AgNP concentrations further supports the protective contribution of its capsular layer. As nanoparticle concentrations increased, however, sufficient quantities of AgNPs accumulated around bacterial cells to overcome this structural defense, resulting in progressively larger zones of inhibition. These findings indicate that the antibacterial effectiveness of AgNPs depends not only on nanoparticle characteristics but also on the intrinsic structural features of the target microorganism.

Another noteworthy finding was the comparatively weak antibacterial activity displayed by the crude *S. costus* extract alone. Following nanoparticle synthesis, antimicrobial potency increased substantially, indicating that conversion of silver ions into nanoscale particles markedly enhanced the biological activity of the plant extract. Formation of AgNPs dramatically increases the reactive surface area while simultaneously concentrating bioactive phytochemicals on the nanoparticle surface. Consequently, the observed antibacterial activity is likely the result of synergistic interactions between metallic silver and phytochemical constituents comprising the natural surface corona. Similar synergistic enhancement has been widely reported for medicinal plant-mediated AgNPs, where biosynthesized nanoparticles exhibit considerably greater antibacterial efficacy than either crude extracts or silver salts used individually [45,46].

The present findings are also in agreement with recent advances in eco-friendly nanomaterial synthesis for combating multidrug-resistant pathogens. Jawad et al. reported that green-synthesized MgS@CuS nanocomposites exhibited promising antibacterial activity against multidrug-resistant bacteria, highlighting the potential of environmentally friendly nanomaterials as alternative antimicrobial agents. These observations further support the growing application of green nanotechnology for overcoming antimicrobial resistance and emphasize the therapeutic potential of biogenic nanomaterials in the management of drug-resistant bacterial infections.

Although the biosynthesized AgNPs exhibited potent antibacterial activity against the tested bacterial strains, the precise molecular mechanism responsible for bacterial killing was not directly investigated in the present study. Based on previous reports, the antibacterial activity of AgNPs is generally attributed to multiple complementary mechanisms, including adhesion to the bacterial cell envelope, disruption of membrane integrity, enhanced membrane permeability, intracellular release of Ag^+^ ions, excessive reactive oxygen species (ROS) generation, oxidative damage to proteins, lipids, and nucleic acids, interference with respiratory enzymes, ATP depletion, and inhibition of DNA replication. These interconnected processes ultimately result in irreversible cellular damage and bacterial death. Nevertheless, the contribution of each mechanism to the antibacterial activity of the *Saussurea costus*-mediated AgNPs remains to be experimentally verified.^31^ Future studies should therefore combine antibacterial susceptibility testing with mechanistic assays such as intracellular ROS quantification, membrane permeability and depolarization analyses, scanning and transmission electron microscopy of treated bacterial cells, ATP and protein leakage measurements, oxidative stress biomarker evaluation, DNA damage assays, and intracellular silver accumulation analysis. Such investigations would provide direct mechanistic evidence and improve the understanding of the bactericidal action of the biosynthesized AgNPs.

The antibacterial activity observed in the present study was comparable to that reported for several recently published plant-mediated AgNPs against multidrug-resistant Gram-negative bacteria. However, differences in inhibition zone diameters, MIC, and MBC values among studies should be interpreted cautiously because antibacterial performance is influenced by nanoparticle size, morphology, surface chemistry, capping phytochemicals, bacterial strain characteristics, inoculum density, and experimental methodology. In general, smaller nanoparticles with larger specific surface areas exhibit enhanced interaction with bacterial membranes and greater antimicrobial efficacy, which may partially explain the variability reported in the literature.

### 4.6 MIC and MBC demonstrate potent bactericidal activity

The broth microdilution assay further confirmed the potent antimicrobial activity of the synthesized nanoparticles. Both MIC and MBC values were relatively low, indicating that only small concentrations of AgNPs were sufficient to inhibit bacterial growth and eliminate viable bacterial populations. Moreover, the close agreement between MIC and MBC values suggests that the nanoparticles primarily exerted a bactericidal rather than bacteriostatic effect.^32^

This distinction has important clinical implications because bactericidal agents directly destroy bacterial cells, whereas bacteriostatic compounds merely suppress bacterial multiplication and rely on host immune defenses for complete eradication of infection. In the management of infections caused by multidrug-resistant bacteria, especially among immunocompromised individuals, bactericidal activity is generally considered more desirable because it accelerates bacterial clearance and reduces the likelihood of persistent infection or emergence of additional resistance mechanisms.^3^

The favorable MIC values obtained in this investigation compare well with those reported for numerous other medicinal plant-derived AgNPs. This enhanced antibacterial potency is likely attributable to the physicochemical properties identified during nanoparticle characterization, including their relatively small size, pronounced crystallinity, narrow particle-size distribution, stable phytochemical coating, and satisfactory colloidal stability. Collectively, these characteristics facilitate efficient interactions between nanoparticles and bacterial cells, thereby reducing the concentration required to achieve complete inhibition of bacterial growth.^33^

The effectiveness of the synthesized AgNPs against ESBL-producing clinical isolates is particularly significant because these pathogens possess multiple resistance mechanisms, including β-lactamase production, reduced membrane permeability, active efflux pumps, and biofilm-forming capacity. Despite these defense strategies, the nanoparticles retained strong antibacterial activity, supporting the concept that AgNP-mediated killing circumvents many conventional antibiotic resistance pathways. Since silver nanoparticles simultaneously target multiple cellular structures and metabolic processes instead of a single molecular site, bacteria have considerably fewer opportunities to develop stable resistance compared with traditional antimicrobial agents.^24^ Direct comparison of MIC values among different studies is difficult because synthesis protocols, bacterial strains, inoculum density, and testing conditions vary considerably.

### 4.7 Time-kill kinetics reveal rapid and sustained bacterial elimination

Although MIC and MBC assays provide valuable information regarding the inhibitory and bactericidal concentrations of an antimicrobial agent, they represent only endpoint measurements and do not describe the dynamics of bacterial killing. In contrast, the time-kill assay performed in the present study demonstrated that exposure to *S. costus*-mediated AgNPs resulted in a progressive decline in bacterial viability over time. Moreover, bacterial killing became increasingly pronounced as nanoparticle concentrations increased from MIC to 2 × MIC and 4 × MIC. This concentration-dependent reduction in viable bacterial counts indicates that AgNP-induced cell death occurs through cumulative cellular damage rather than an immediate lethal event.^25^

The rapid decrease in bacterial survival observed during the initial phase of treatment is likely attributable to the prompt interaction between AgNPs and the bacterial outer membrane. Following attachment to the cell surface, silver nanoparticles and the Ag^+^ ions released from them destabilize membrane integrity through electrostatic interactions, leading to increased membrane permeability, leakage of intracellular constituents, disruption of membrane potential, and impairment of membrane-associated respiratory enzymes. Continued exposure subsequently promotes intracellular accumulation of silver ions, resulting in excessive production of reactive oxygen species (ROS), oxidation of cellular proteins, ribosomal dysfunction, DNA damage, and ultimately irreversible bacterial death.^25^

Another important observation was the maintenance of antibacterial activity throughout the 24-h incubation period. Rather than releasing large amounts of silver ions immediately after exposure, the phytochemical-coated nanoparticles appeared to function as a sustained reservoir capable of gradually liberating bioactive Ag^+^ ions over time. Such controlled ion release is advantageous for biomedical applications because it prolongs antimicrobial efficacy while minimizing unnecessary exposure of surrounding healthy tissues to excessive silver concentrations. The phytochemical corona originating from *S. costus* therefore may contribute not only to nanoparticle stabilization but also to regulation of silver ion release kinetics, thereby enhancing both antibacterial effectiveness and biocompatibility.^34^

Collectively, the findings obtained from the agar diffusion assay, MIC, MBC, and time-kill analyses consistently demonstrate the potent bactericidal activity of *S. costus*-mediated AgNPs against clinically relevant ESBL-producing pathogens. The strong agreement among these independent microbiological methods further reinforces the reliability of the present findings and supports the potential use of these biosynthesized nanoparticles as promising antimicrobial nanomaterials for the management of multidrug-resistant bacterial infections.^11^

The time-kill assay demonstrated a clear concentration-dependent bactericidal effect of the biosynthesized AgNPs, indicating that higher nanoparticle concentrations accelerated bacterial killing. However, the present analysis was limited to qualitative evaluation of bacterial survival over time and did not include quantitative pharmacodynamic modeling. Future studies should incorporate log10 CFU reduction analysis, estimation of bactericidal rate constants, concentration–time response modeling, and comparison with conventional antibiotics to characterize the killing kinetics more comprehensively. Such quantitative analyses would facilitate determination of concentration-dependent versus time-dependent antibacterial behavior and provide a stronger basis for optimizing therapeutic dosing strategies in future biomedical applications.

### 4.8 Proposed antibacterial mechanism of *Saussurea costus*-mediated AgNPs

Growing evidence indicates that the antimicrobial activity of silver nanoparticles is not mediated through a single pathway but instead results from multiple complementary mechanisms acting simultaneously. Accordingly, the pronounced antibacterial activity demonstrated by *S. costus*-mediated AgNPs against ESBL-producing *E. coli* and *K. pneumoniae* in the present study is most likely attributable to the combined action of several physicochemical and biochemical processes that collectively overwhelm bacterial defense systems.

The antibacterial process is initiated by rapid adsorption of AgNPs onto the bacterial surface. Gram-negative bacteria possess outer membranes rich in lipopolysaccharides (LPS), phospholipids, and membrane proteins containing negatively charged functional groups that facilitate interaction with silver nanoparticles and released Ag^+^ ions. Following surface attachment, AgNPs disrupt membrane organization, increase membrane permeability, and compromise membrane integrity. These structural alterations lead to leakage of intracellular contents, collapse of membrane potential, disruption of the proton motive force, and inhibition of ATP production. Previous electron microscopic studies have consistently demonstrated membrane deformation, pore formation, and eventual collapse of bacterial cells after treatment with biosynthesized AgNPs, supporting this initial mechanism of action.^18^

Following membrane damage, silver ions released from the nanoparticle surface penetrate into the bacterial cytoplasm and interact with multiple intracellular targets. Owing to their strong affinity for sulfhydryl (-SH) groups, Ag^+^ ions readily bind to numerous enzymes involved in essential metabolic pathways, including cellular respiration, oxidative phosphorylation, amino acid biosynthesis, and DNA replication. Inactivation of these enzymes progressively disrupts bacterial metabolism, impairs energy generation, and ultimately compromises cellular homeostasis.

In addition to metabolic inhibition, AgNPs interfere directly with bacterial genetic processes. Silver ions interact with phosphate groups and nitrogenous bases within DNA, thereby suppressing DNA replication and transcription. Simultaneously, interactions with ribosomal proteins impair protein synthesis, preventing bacteria from replacing damaged structural and enzymatic proteins. The simultaneous disruption of DNA replication, RNA transcription, and protein translation severely limits the ability of bacterial cells to recover following initial membrane injury, ultimately leading to irreversible cell death.^23^

Another important characteristic of AgNPs is their ability to interfere with bacterial communication systems and biofilm development. Biofilm formation represents one of the major survival strategies employed by ESBL-producing pathogens because the extracellular polymeric matrix limits antimicrobial penetration while facilitating horizontal transfer of resistance genes. Previous investigations have demonstrated that silver nanoparticles inhibit quorum sensing, suppress extracellular polysaccharide production, and interfere with biofilm maturation, thereby increasing bacterial susceptibility to antimicrobial agents (Singh et al., 2024). Although biofilm formation was not directly evaluated in the present investigation, the potent bactericidal activity observed against highly resistant clinical isolates suggests that inhibition of biofilm-associated processes may also contribute to the antibacterial effects of the synthesized nanoparticles.

The phytochemical corona surrounding the biosynthesized AgNPs is also likely to play an active biological role beyond simply stabilizing the nanoparticles. FTIR analysis confirmed that flavonoids, polyphenols, proteins, and other plant-derived metabolites remained associated with the nanoparticle surface after synthesis. Many of these compounds possess intrinsic antimicrobial properties through mechanisms including membrane disruption, enzyme inhibition, and metal chelation. Their presence may therefore enhance nanoparticle attachment to bacterial cells, improve nanoparticle dispersion, and regulate the gradual release of silver ions. Such synergistic interactions between metallic silver and phytochemicals are increasingly recognized as a major advantage of green-synthesized nanoparticles over chemically produced counterparts.

Importantly, the antibacterial mechanisms described above differ fundamentally from those targeted by conventional antibiotics. While ESBL enzymes effectively hydrolyze β-lactam antibiotics, they are incapable of inactivating metallic nanoparticles or silver ions. Likewise, resistance mechanisms such as altered penicillin-binding proteins, antibiotic-modifying enzymes, or multidrug efflux pumps provide only limited protection against the simultaneous membrane disruption, oxidative stress, metabolic inhibition, and nucleic acid damage induced by AgNPs. Consequently, bacteria are considerably less likely to acquire resistance against the multiple concurrent mechanisms employed by silver nanoparticles than against antibiotics directed toward a single molecular target.

Overall, the available evidence suggests that *S. costus*-mediated AgNPs function as multitarget antimicrobial nanomaterials capable of simultaneously damaging bacterial membranes, inducing oxidative stress, inhibiting key metabolic pathways, interfering with nucleic acid synthesis, and suppressing biofilm-associated defense mechanisms. This integrated mode of action provides a mechanistic explanation for the strong bactericidal activity observed in the present study and further highlights the therapeutic potential of green-synthesized AgNPs as alternative antimicrobial agents for combating multidrug-resistant bacterial infections.

Our findings are consistent with those of Yosif et al. (2024), who reported that nanoparticle-based formulations effectively inhibited biofilm formation and disrupted mature biofilms of multidrug-resistant *K. pneumoniae*, highlighting the potential of nanomaterials as alternative antibiofilm agents.^35^

The apparent discrepancy between the concentrations used in the agar well diffusion assay and the MIC values reflects the different principles underlying these methods. In broth microdilution, AgNPs remain uniformly suspended and continuously interact with bacterial cells, allowing inhibition at relatively low concentrations. In contrast, the agar well diffusion assay depends on passive diffusion of nanoparticles through a semi-solid agar matrix, which is substantially restricted by nanoparticle size, hydrodynamic diameter, aggregation, and interactions with agar components. Consequently, higher concentrations are generally required to produce measurable inhibition zones. Therefore, inhibition zone diameters should not be interpreted as directly equivalent to MIC values. Importantly, the inhibition zones observed at 12.5 μg/mL (9.8 and 8.5 mm) remain biologically meaningful because they demonstrate detectable antibacterial activity at concentrations close to the MIC. Their relatively small diameters primarily reflect diffusion limitations rather than weak intrinsic antimicrobial activity.

### 4.9 Cytocompatibility of *Saussurea costus*-mediated AgNPs

For antimicrobial nanomaterials to be considered suitable for biomedical applications, they must exhibit not only effective antibacterial activity but also an acceptable level of compatibility with mammalian cells. Accordingly, the cytocompatibility of the biosynthesized *Saussurea costus*-mediated AgNPs was evaluated using human dermal fibroblast adult (HDFa) cells through the MTT assay. The obtained results revealed a concentration-dependent decrease in cell viability after 24 h of exposure. Nevertheless, low and intermediate nanoparticle concentrations maintained cell viability above the level generally regarded as acceptable for in vitro biocompatibility, whereas only the highest tested concentration induced moderate cytotoxic effects. These observations indicate that the synthesized AgNPs possess a favorable balance between antimicrobial efficacy and mammalian cell compatibility.^36^

The gradual decline in cell viability observed with increasing nanoparticle concentration is consistent with previous reports describing the biological behavior of plant-mediated silver nanoparticles. At relatively low concentrations, phytochemical-coated AgNPs generally exert minimal adverse effects on mammalian cells. However, as nanoparticle concentration increases, enhanced cellular uptake and intracellular accumulation occur, leading to progressive impairment of mitochondrial metabolic activity, which is reflected by reduced MTT assay readings. Similar dose-dependent cytotoxic responses have been documented for AgNPs synthesized using various medicinal plant extracts, highlighting nanoparticle concentration as one of the principal determinants of cellular response.^14^

The relatively favorable cytocompatibility demonstrated in the present study is likely associated with the green synthesis strategy employed. FTIR characterization confirmed that the nanoparticle surface remained coated with phytochemicals originating from *S. costus* root extract, including flavonoids, phenolic compounds, and other oxygen-containing biomolecules. Besides functioning as stabilizing agents, these naturally occurring surface constituents likely reduce direct interactions between the metallic silver core and mammalian cell membranes, thereby improving biocompatibility relative to chemically synthesized AgNPs prepared with synthetic reducing and stabilizing agents. Similar protective effects attributed to phytochemical surface coatings have been reported in numerous previous studies investigating green-synthesized silver nanoparticles.^14^

The physicochemical characteristics of the synthesized nanoparticles may also have contributed to their favorable biological behavior. TEM analysis demonstrated that the nanoparticles were relatively small and predominantly spherical, whereas DLS and zeta potential measurements confirmed satisfactory colloidal stability in aqueous media. These characteristics facilitate homogeneous nanoparticle dispersion while minimizing irreversible aggregation, thereby reducing localized accumulation of nanoparticles on cultured cells. Previous investigations have consistently shown that particle size, morphology, surface chemistry, and colloidal stability collectively influence nanoparticle–cell interactions and play important roles in determining cytocompatibility.^34^

A particularly important finding of this investigation is the simultaneous observation of potent antibacterial activity and acceptable cytocompatibility within the tested concentration range. Although the synthesized AgNPs effectively inhibited ESBL-producing *Escherichia coli* and *Klebsiella pneumoniae*, they produced only limited cytotoxic effects on HDFa cells at concentrations exhibiting antimicrobial activity. This selective biological response may be explained by the greater susceptibility of bacterial cells to nanoparticle-induced membrane disruption, oxidative stress, and metabolic inhibition compared with mammalian cells, which possess more complex membrane structures together with more efficient cellular defense and repair mechanisms.^36^

It should be recognized that the MTT assay primarily assesses mitochondrial metabolic activity and therefore provides only an initial estimate of in vitro cytocompatibility rather than a comprehensive evaluation of nanoparticle biosafety. Accordingly, additional investigations examining apoptosis, oxidative stress markers, inflammatory responses, hemocompatibility, biodistribution, and long-term in vivo toxicity are required before the clinical safety profile of *S. costus*-mediated AgNPs can be fully established.^37^

Overall, the findings demonstrate that biosynthesized *S. costus*-mediated AgNPs combine acceptable cytocompatibility with strong antibacterial activity against multidrug-resistant ESBL-producing pathogens. These characteristics support their potential application as promising antimicrobial nanomaterials for future biomedical and pharmaceutical applications.

The cytocompatibility of the biosynthesized AgNPs was evaluated using primary human dermal fibroblasts (HDFa) as an initial model to determine their potential suitability for biomedical applications. Although the results demonstrated acceptable compatibility with HDFa cells within the investigated concentration range, evaluation in a single cell type does not fully represent the complexity of human tissues. Future studies should therefore investigate the biological responses of additional clinically relevant cell types, including keratinocytes, macrophages, endothelial cells, and epithelial cells, to obtain a broader assessment of biocompatibility.

Furthermore, the present study primarily assessed cell viability and did not investigate the molecular mechanisms underlying nanoparticle-induced cellular responses. Comprehensive toxicological evaluation should include apoptosis and necrosis assays, intracellular oxidative stress measurements, inflammatory cytokine profiling, mitochondrial function analysis, and DNA damage assessment. Such mechanistic studies would provide a more complete understanding of the biological safety of *Saussurea costus*-mediated AgNPs and facilitate their potential translation toward biomedical applications.

Although both antibacterial activity and cytocompatibility were evaluated in the present study, a quantitative selectivity index (SI) was not determined because IC₅₀/CC₅₀ values were not established under the experimental conditions employed. Future studies should integrate detailed cytotoxicity analyses with antibacterial susceptibility testing to calculate SI values, thereby providing a quantitative assessment of the therapeutic window and facilitating comparison with other antimicrobial nanomaterials.

The favorable cytocompatibility observed in HDFa cells is generally consistent with previous reports demonstrating that plant-mediated AgNPs often exhibit lower cytotoxicity than chemically synthesized nanoparticles. This effect has been attributed to the presence of phytochemical capping molecules that may reduce direct nanoparticle–cell interactions and improve colloidal stability. Nevertheless, differences in cell type, nanoparticle concentration, exposure duration, and synthesis methodology make direct comparison between studies challenging.

### 4.10 Overall interpretation of the findings

The results of the present investigation collectively demonstrate that *Saussurea costus* root extract can be successfully utilized for the green synthesis of silver nanoparticles possessing desirable physicochemical and biological characteristics. Comprehensive characterization confirmed the formation of stable, crystalline, and predominantly spherical AgNPs with nanoscale dimensions and favorable colloidal stability. In addition, FTIR analysis demonstrated that phytochemicals derived from the plant extract remained associated with the nanoparticle surface, indicating that the same biomolecules responsible for reducing silver ions also served as natural stabilizing agents throughout the biosynthetic process.^38^

Biological evaluation consistently confirmed the potent antibacterial activity of the synthesized nanoparticles against ESBL-producing *Escherichia coli* and *Klebsiella pneumoniae*. The concentration-dependent inhibition observed in the agar well diffusion assay, together with the low MIC and MBC values and the rapid bactericidal activity demonstrated by the time-kill experiments, collectively indicate that the biosynthesized AgNPs efficiently suppressed the growth and survival of clinically relevant multidrug-resistant bacteria. These microbiological findings are fully supported by the favorable physicochemical characteristics identified during nanoparticle characterization, particularly their relatively small particle size, pronounced crystallinity, and stable phytochemical surface coating, all of which are known to promote efficient nanoparticle–bacteria interactions.^32^

Evaluation of cytocompatibility further demonstrated that the biosynthesized nanoparticles maintained acceptable compatibility with HDFa cells across the investigated concentration range. These findings suggest that the green synthesis approach generated AgNPs capable of combining strong antimicrobial activity with satisfactory mammalian cell safety. The phytochemical corona derived from *S. costus* likely played an important role in minimizing undesirable interactions with mammalian cells while preserving the antimicrobial effectiveness of the silver nanoparticles.^39^

### 4.11 Strengths of the study

The present study demonstrates several important strengths. First, an environmentally friendly and sustainable green synthesis approach was successfully employed using *Saussurea costus* extract as both the reducing and stabilizing agent, thereby avoiding hazardous chemical reagents. Second, the biosynthesized AgNPs were comprehensively characterized using complementary physicochemical techniques, including UV–Vis spectroscopy, FTIR, XRD, TEM, DLS, and zeta potential analysis, providing evidence for successful nanoparticle formation and stability. Third, the nanoparticles exhibited significant antibacterial activity against clinically relevant ESBL-producing bacterial isolates together with acceptable cytocompatibility toward primary human dermal fibroblasts (HDFa). Finally, the integration of physicochemical characterization with biological evaluation provides a useful foundation for the future development of plant-mediated antimicrobial nanomaterials.

### 4.12 Limitations

Although FTIR analysis demonstrated the presence of hydroxyl, carbonyl, and other oxygen-containing functional groups associated with the biosynthesized AgNPs, it does not provide definitive molecular identification of the phytochemicals adsorbed on the nanoparticle surface. Therefore, the proposed roles of phenolics, flavonoids, terpenoids, and other metabolites in Ag^+^ reduction and nanoparticle stabilization should be considered mechanistically plausible but indirectly supported by the present physicochemical evidence. More comprehensive surface characterization using X-ray photoelectron spectroscopy (XPS), Raman spectroscopy, and detailed phytochemical profiling by LC-MS/MS or GC–MS would enable identification of the specific compounds involved in electron transfer, surface binding, and stabilization. These advanced analytical approaches represent an important direction for future investigations and would further strengthen the mechanistic understanding of *Saussurea costus*-mediated AgNP biosynthesis.

Although the biosynthesized AgNPs exhibited good initial colloidal stability as indicated by their hydrodynamic size distribution and zeta potential, the present study did not investigate long-term physicochemical stability under storage or physiological conditions. Because nanoparticle aggregation and surface modification may occur over time or in complex biological environments, further studies should evaluate colloidal stability during prolonged storage and under physiologically relevant conditions, including different pH values, saline solutions, protein-containing media, serum, and cell culture media. Such investigations would provide valuable information regarding aggregation behavior, protein corona formation, and changes in particle size, surface charge, and biological activity, thereby facilitating the translation of biosynthesized AgNPs toward biomedical applications.

Although all bacterial isolates included in this study were phenotypically confirmed as ESBL producers using standard microbiological methods, the underlying resistance genes were not molecularly characterized. Consequently, the antibacterial activity of the biosynthesized AgNPs could not be correlated with specific β-lactamase genotypes such as blaCTX-M, blaTEM, blaSHV, blaNDM, or blaOXA. Future studies should combine phenotypic antimicrobial susceptibility testing with molecular characterization of resistance determinants using PCR or whole-genome sequencing to determine whether nanoparticle susceptibility varies among different resistance genotypes. Such an integrated approach would improve the clinical relevance of biosynthesized AgNPs as potential therapeutic agents against multidrug-resistant bacterial pathogens.

### 4.13 Future perspectives

Although the biosynthesized AgNPs exhibited potent antibacterial activity against planktonic ESBL-producing bacterial isolates, their effects on bacterial biofilms were not evaluated in the present study. Biofilm formation represents an important survival strategy that enhances antimicrobial tolerance and contributes to the persistence of chronic infections. Therefore, the antibacterial efficacy demonstrated against planktonic bacteria cannot be directly extrapolated to biofilm-associated infections. Future investigations should evaluate both biofilm inhibition and biofilm eradication using complementary techniques such as crystal violet biomass quantification, confocal laser scanning microscopy (CLSM), extracellular polymeric substance (EPS) analysis, live/dead fluorescence staining, and determination of the minimum biofilm eradication concentration (MBEC). Such studies would provide a more comprehensive assessment of the therapeutic potential of *Saussurea costus*-mediated AgNPs against clinically relevant biofilm-associated infections.

Although the present study demonstrated promising antibacterial activity and favorable cytocompatibility under in vitro conditions, these findings should be considered as preliminary evidence of biomedical potential rather than confirmation of clinical applicability. Translation of biosynthesized AgNPs into biomedical use requires comprehensive in vivo evaluation to determine therapeutic efficacy, biological safety, and systemic behavior. Future studies should therefore investigate the performance of *Saussurea costus*-mediated AgNPs in appropriate animal models, including murine wound infection and systemic infection models. In addition, detailed toxicological evaluation, biodistribution analysis, pharmacokinetic profiling, organ accumulation studies, and histopathological examination will be necessary to establish the safety profile and therapeutic feasibility of the biosynthesized nanoparticles prior to clinical translation.

Although the present study demonstrates the successful biosynthesis of biologically active AgNPs using *Saussurea costus* extract, it does not include a direct experimental comparison with chemically synthesized AgNPs prepared under identical conditions. Consequently, the relative advantages of the green synthesis approach with respect to antibacterial activity, antioxidant properties, colloidal stability, and cytocompatibility cannot be conclusively established. Nevertheless, previous studies have suggested that plant-mediated AgNPs often exhibit improved colloidal stability and reduced cytotoxicity owing to the presence of phytochemical capping agents, while maintaining comparable or enhanced antimicrobial activity. However, such comparisons should be interpreted cautiously because differences in synthesis protocols, particle size, morphology, surface chemistry, and experimental conditions may substantially influence biological performance. Future studies should therefore perform direct side-by-side comparisons between chemically synthesized and plant-mediated AgNPs using identical characterization methods and biological assays to establish the genuine advantages of green synthesis.

Despite the promising antibacterial activity and favorable physicochemical characteristics of the biosynthesized AgNPs, several important challenges remain before clinical or industrial translation can be achieved. One of the major limitations of plant-mediated nanoparticle synthesis is the potential batch-to-batch variability resulting from differences in phytochemical composition caused by plant genotype, geographical origin, harvesting season, environmental conditions, extraction procedures, and storage conditions. Such variability may influence the reduction efficiency, particle size distribution, surface chemistry, and biological activity of the synthesized nanoparticles, thereby affecting reproducibility.

In addition, large-scale production of biosynthesized nanoparticles requires standardized extraction protocols, reproducible manufacturing processes, and rigorous quality-control procedures to ensure consistent physicochemical properties between production batches. Industrial implementation will also require optimization of process scalability while maintaining nanoparticle stability, purity, and biological performance. From a regulatory perspective, clinical translation of plant-mediated AgNPs will require compliance with Good Manufacturing Practice (GMP), standardized characterization methods, comprehensive toxicological evaluation, long-term stability assessment, and well-defined manufacturing specifications to satisfy regulatory requirements. Therefore, although the present study demonstrates the feasibility of producing biologically active AgNPs using *Saussurea costus* extract, additional studies addressing standardization, reproducibility, industrial manufacturing, and regulatory compliance will be essential before practical biomedical application can be realized.

In addition to biomedical safety, the potential environmental impact of silver nanoparticles should also be considered prior to their large-scale production and application. Although plant-mediated synthesis reduces the use of hazardous chemical reducing agents and is generally regarded as a more environmentally friendly manufacturing approach, the resulting AgNPs may still pose ecological risks following environmental release. Silver nanoparticles have been reported to persist in environmental matrices and may exhibit toxicity toward aquatic organisms, accumulate in soil, and alter the structure and function of beneficial microbial communities involved in nutrient cycling and ecosystem stability.

Furthermore, prolonged or uncontrolled environmental exposure to AgNPs may contribute to the selection of silver-tolerant microorganisms and potentially promote co-selection of antimicrobial resistance determinants. Therefore, careful evaluation of nanoparticle fate, transport, transformation, biodegradation, and ecotoxicological effects under environmentally relevant conditions is necessary. In addition, appropriate waste management strategies and disposal protocols should be established to minimize unintended environmental exposure during laboratory research, industrial manufacturing, and clinical use. Consequently, future investigations should integrate environmental risk assessment with biomedical evaluation to ensure the responsible and sustainable development of plant-mediated silver nanoparticles.

### 4.14 Sustainable development goals (SDGs) relevance

The present study supports several United Nations Sustainable Development Goals (SDGs). The development of plant-mediated silver nanoparticles with antibacterial activity contributes to SDG 3 (Good Health and Well-being) by providing environmentally sustainable approaches for combating antimicrobial-resistant pathogens. The green synthesis strategy, which minimizes the use of hazardous chemical reducing agents and promotes renewable plant resources, aligns with SDG 12 (Responsible Consumption and Production) through more sustainable manufacturing practices. In addition, the environmentally friendly synthesis methodology supports SDG 9 (Industry, Innovation and Infrastructure) by encouraging the development of innovative and sustainable nanotechnologies. Nevertheless, further investigations addressing environmental safety, nanoparticle standardization, regulatory compliance, and scalable production are required to ensure the responsible implementation of this technology in accordance with sustainable development principles.

Taken together, the results obtained in this study demonstrate that *S. costus*-mediated AgNPs possess an advantageous combination of favorable physicochemical properties, potent antibacterial activity, and acceptable in vitro cytocompatibility. Collectively, these findings reinforce the growing evidence supporting plant-mediated silver nanoparticles as promising alternative or adjunct antimicrobial agents for the treatment of infections caused by ESBL-producing multidrug-resistant bacteria. Nevertheless, additional investigations focusing on molecular mechanisms of action, pharmacokinetic behavior, long-term toxicological evaluation, and validation in appropriate animal infection models remain essential before clinical translation of these nanomaterials can be considered.

## 5 Conclusions

The present study demonstrates that *Saussurea costus* extract can be successfully utilized as a green reducing and stabilizing agent for the biosynthesis of silver nanoparticles with favorable physicochemical characteristics, significant in vitro antibacterial activity against ESBL-producing bacterial isolates, and acceptable cytocompatibility toward human dermal fibroblasts under the investigated experimental conditions. These findings support the potential of plant-mediated AgNPs as promising antimicrobial nanomaterials and contribute to the growing body of research on environmentally friendly nanoparticle synthesis.

Nevertheless, the present findings should be interpreted within the limitations of this study. The proposed antibacterial and nanoparticle synthesis mechanisms were not directly validated experimentally, long-term physicochemical stability under physiological conditions was not investigated, and comprehensive molecular characterization of nanoparticle surface chemistry was not performed. Furthermore, the biological evaluation was limited to in vitro experiments and did not include pharmacokinetic analyses, biodistribution studies, comprehensive toxicological assessment, or in vivo efficacy evaluation. Consequently, additional mechanistic, preclinical, and translational investigations will be necessary before the biomedical or clinical applicability of the biosynthesized AgNPs can be established.

Overall, this study provides a foundation for future research aimed at optimizing plant-mediated silver nanoparticles and evaluating their safety, efficacy, reproducibility, and translational potential through standardized mechanistic studies, comprehensive biological assessment, and well-designed preclinical investigations.

## CRediT authorship contribution statement

**Rebwar Muhammad Hamasalih:** Writing – review & editing, Visualization, Resources, Investigation, Conceptualization.

## Funding

None received

## Declaration of competing interest

The authors declare that they have no known competing financial interests or personal relationships that could have appeared to influence the work reported in this paper.
