# Supplementary material for: Green synthesis of Saussurea costus-mediated silver nanoparticles: Characterization, antibacterial activity against ESBL-producing bacteria, and cytocompatibility
Source: J Genet Eng Biotechnol. 2026 Aug 18;24(4):100794. doi: 10.1016/j.jgeb.2026.100794 (PMC13519643; doi:10.1016/j.jgeb.2026.100794)
Supplement: Supplementary file 1 [file mmc1.docx]

**Supplementary1**. The antimicrobial agents included in the present study

| **Antibiotic** | **Disk Potency** |
| --- | --- |
| Ampicillin | 10 μg |
| Amoxicillin/Clavulanic acid | 20/10 μg |
| Piperacillin/Tazobactam | 100/10 μg |
| Cefotaxime | 30 μg |
| Ceftazidime | 30 μg |
| Ceftriaxone | 30 μg |
| Cefepime | 30 μg |
| Aztreonam | 30 μg |
| Imipenem | 10 μg |
| Meropenem | 10 μg |
| Gentamicin | 10 μg |
| Amikacin | 30 μg |
| Ciprofloxacin | 5 μg |
| Levofloxacin | 5 μg |
| Trimethoprim/Sulfamethoxazole | 1.25/23.75 μg |
| Nitrofurantoin* | 300 μg |

*Nitrofurantoin was tested only for urinary isolates.

**Supplementary 2**. Classification of bacteria based on the biofilm type

| **Biofilm category** | **Optical density** |
| --- | --- |
| Non-biofilm producer | OD ≤ ODc |
| Weak biofilm producer | ODc < OD ≤ 2×ODc |
| Moderate biofilm producer | 2×ODc < OD ≤ 4×ODc |
| Strong biofilm producer | OD > 4×ODc |

Supplementary 4, This image is related to the figure 1 in main manuscript.
